# Evolutionary history of burrowing asps (Lamprophiidae: Atractaspidinae) with emphasis on fang evolution and prey selection

**DOI:** 10.1371/journal.pone.0214889

**Published:** 2019-04-17

**Authors:** Frank Portillo, Edward L. Stanley, William R. Branch, Werner Conradie, Mark-Oliver Rödel, Johannes Penner, Michael F. Barej, Chifundera Kusamba, Wandege M. Muninga, Mwenebatu M. Aristote, Aaron M. Bauer, Jean-François Trape, Zoltán T. Nagy, Piero Carlino, Olivier S. G. Pauwels, Michele Menegon, Ivan Ineich, Marius Burger, Ange-Ghislain Zassi-Boulou, Tomáš Mazuch, Kate Jackson, Daniel F. Hughes, Mathias Behangana, Eli Greenbaum

**Affiliations:** 1 Department of Biological Sciences, University of Texas at El Paso, El Paso, Texas, United States of America; 2 Florida Museum of Natural History, University of Florida, Gainesville, Florida, United States of America; 3 Port Elizabeth Museum, Humewood, South Africa; 4 Department of Zoology, Nelson Mandela University, Port Elizabeth, South Africa; 5 School of Natural Resource Management, George Campus, Nelson Mandela University, George, South Africa; 6 Museum für Naturkunde, Leibniz Institute for Evolution and Biodiversity Science, Berlin, Germany; 7 Department of Wildlife Ecology and Wildlife Management, University of Freiburg, Freiburg, Germany; 8 Laboratoire d’Herpétologie, Département de Biologie, Centre de Recherche en Sciences Naturelles, Lwiro, South Kivu, Democratic Republic of the Congo; 9 Institut Supérieur d'Écologie pour la Conservation de la Nature, Katana Campus, South Kivu, Democratic Republic of the Congo; 10 Department of Biology, Villanova University, Villanova, Pennsylvania, United States of America; 11 Laboratoire de Paludologie et Zoologie Médicale, Institut de Recherche pour le Développement, Dakar, Senegal; 12 Independent Researcher, Berlin, Germany; 13 Museo di Storia naturale del Salento, Calimera, Italy; 14 Département des Vertébrés Récents, Institut Royal des Sciences naturelles de Belgique, Brussels, Belgium; 15 Division of Biology and Conservation Ecology, School of Science and the Environment, Manchester Metropolitan University, Manchester, United Kingdom; 16 Muséum National d’Histoire Naturelle, Sorbonne Universités, Département Systématique et Evolution (Reptiles), ISyEB (Institut de Systématique, Évolution, Biodiversité), Paris, France; 17 African Amphibian Conservation Research Group, Unit for Environmental Sciences and Management, North-West University, Potchefstroom, South Africa; 18 Flora Fauna & Man, Ecological Services Ltd. Tortola, British Virgin Islands; 19 Institut National de Recherche en Sciences Exactes et Naturelles, Brazzaville, Republic of Congo; 20 Independent Researcher, Dříteč, Czech Republic; 21 Department of Biology, Whitman College, Walla Walla, Washington, United States of America; 22 Department of Environmental Sciences, Makerere University, Kampala, Uganda; State Museum of Natural History, GERMANY

## Abstract

Atractaspidines are poorly studied, fossorial snakes that are found throughout Africa and western Asia, including the Middle East. We employed concatenated gene-tree analyses and divergence dating approaches to investigate evolutionary relationships and biogeographic patterns of atractaspidines with a multi-locus data set consisting of three mitochondrial (*16S*, *cyt b*, and *ND4*) and two nuclear genes (*c-mos* and *RAG1*). We sampled 91 individuals from both atractaspidine genera (*Atractaspis* and *Homoroselaps*). Additionally, we used ancestral-state reconstructions to investigate fang and diet evolution within Atractaspidinae and its sister lineage (Aparallactinae). Our results indicated that current classification of atractaspidines underestimates diversity within the group. Diversification occurred predominantly between the Miocene and Pliocene. Ancestral-state reconstructions suggest that snake dentition in these taxa might be highly plastic within relatively short periods of time to facilitate adaptations to dynamic foraging and life-history strategies.

## 1. Introduction

Recently, several studies generated phylogenies of advanced African snakes, including colubrids, lamprophiids, elapids, and viperids [1–9]. In contrast, there has been only one morphology-based, phylogenetic study that focused on atractaspidines [10]. The Family Atractaspididae was originally erected by Günther [11] for species of *Atractaspis*, renowned for their unique and exceptionally long and mobile fangs [12]. Based on skull morphology, Bourgeois [13] created the subfamily Aparallactinae (within Colubridae) to accommodate *Atractaspis*, *Aparallactus*, and other closely related fossorial snakes. This grouping was supported by jaw musculature studies of Heymans [14–15], who transferred *Atractaspis* to the Subfamily Atractaspidinae (Atractaspininae, *sensu* Kelly et al. [16]). Several recent molecular [7–9] and morphological studies [17–18] recovered a monophyletic group containing both aparallactines and atractaspidines, and with few exceptions [19–21], current classification recognizes Aparallactinae and Atractaspidinae as sister taxa in the Family Lamprophiidae [2, 7–9, 22–25]. Phylogenetic relationships within atractaspidines are not well known, because many phylogenetic studies that included atractaspidines were limited by low sample sizes [2, 8–10, 21–23, 26–27].

Based on scale patterns and counts, Laurent [28] assigned the known species of *Atractaspis* into five groups (Sections A–E). Decades later, Underwood and Kochva [18] partitioned *Atractaspis* into two groups based on venom gland morphology and geographic distribution: the ‘*bibronii*’ group and the ‘*microlepidota’* group. These authors defined the ‘*bibronii*’ group as having normal-sized venom glands and a sub-Saharan distribution, and it included the following species: *A*. *aterrima*, *A*. *bibronii*, *A*. *boulengeri*, *A*. *congica*, *A*. *corpulenta*, *A*. *dahomeyensis*, *A*. *duerdeni*, *A*. *irregularis*, and *A*. *reticulata*. The 2nd ‘*microlepidota*’ group has relatively elongated venom glands and is found in western, central and eastern Africa, including the distinctive horn of Africa, the Sinai Peninsula, and much of Arabia, Israel, and the Levant. This latter group consisted of the following species: *A*. *engaddensis*, *A*. *engdahli*, *A*. *leucomelas*, *A*. *microlepidota*, *A*. *micropholis*, and *A*. *scorteccii*. Moyer and Jackson [10] reconstructed phylogenetic relationships among 14 species of *Atractaspis* with morphological data, incorporating *Macrelaps* and *Homoroselaps* as outgroups, based on previous studies [18]. However, the two groups of Underwood and Kochva [18] were not supported [10]. More recent molecular phylogenetic studies suggest that *Homoroselaps* is sister to *Atractaspis*, whereas *Macrelaps* is closely related to *Amblyodipsas* and *Xenocalamus* [8–9, 27].

The diversification of burrowing asps is particularly interesting because of their unique front fangs, which are starkly different from other lamprophiids [21, 29–32]. It has been hypothesized that foraging for nestling mammalian prey was a major driver in the evolution of front fangs and “side-stabbing,” which are unique to *Atractaspis* [31, 33]. Both *Atractaspis* and *Homoroselaps* have front fangs, which differs from the rear-fang morphology that is common in their aparallactine sister group. Although *Atractaspsis* and *Homoroselaps* both contain front fangs, *Atractaspis* fang morphology is more similar to viperids (*Atractaspis* was previously and erroneously classified in the Viperidae), whereas *Homoroselaps* fang morphology is more similar to elapids [25, 31]. Underwood and Kochva [18] suggested a *Macrelaps*-like ancestor for aparallactines and atractaspidines, which may have foraged above ground and fed on a wide variety of prey items. Specialization on elongated prey items (e.g., squamates and invertebrates) may have taken different evolutionary routes within aparallactines and atractaspidines, which involved morphological changes that facilitated foraging, capture, and envenomation of prey items [31]. Burrowing asps and their sister group Aparallactinae are ideal groups to study fang evolution, because they possess many fang types (i.e., rear fang, fixed front fang, and moveable front fang) [25, 29–32]. Additionally, collared snakes (aparallactines) and burrowing asps make interesting models to study fang evolution because of their dietary specializations, especially prevalent within the Aparallactinae, which feed on prey ranging from earthworms to blind snakes [25, 31].

Herein, we employ phylogenetic hypotheses in conjunction with temporal biogeographic information to gain a more comprehensive understanding of the evolutionary history of Atractaspidinae. Specifically, we evaluate the following questions: Are currently recognized genera and species monophyletic? Are *Atractaspis* and *Homoroselaps* sister taxa? Are *Atractaspis* genetically partitioned into the ‘*bibronii*’ and ‘*microlepidota*’ groups as Underwood and Kochva [18] suggested? Additionally, we investigate patterns of diversification regarding character traits, including prey selection and fang morphology, within atractaspidines and aparallactines.

## 2. Materials and methods

### 2.1 Approvals and permissions

Permission for DFH, MB and EG to collect snakes in Uganda was obtained from the Uganda Wildlife Authority (UWA—permit no. 2888 issued on August 1, 2014, permit no. 29279 issued on August 11, 2015) and the Ministry of Tourism, Wildlife and Antiquities (permit no. GoU/008/2016). Permission for CK, WMM, MMA, and EG to collect snakes in Burundi was granted by the Institut National pour l’Environnement et la Conservation de la Nature (INECN—unnumbered permit from Directeur General de l’INECN dated December 27, 2011). Permission for CK, WMM, MMA, DFH, and EG to collect snakes in Democratic Republic of Congo (DRC) was granted by the Centre de Recherche en Sciences Naturelles (CRSN—LW1/28/BB/MM/BIR/050/07, unnumbered permit from 2008, LWI/27/BBa/MUH.M/BBY/141/09, LWI/27/BBa/MUH.M/BBY/023/10, LWI/27/BBa/MUH.M/BBY/001/011, LWI/27/BBa/CIEL/BBY/003/012, LW1/27/BB/KB/BBY/60/2014, LWI/27/BBa/BBY/146/014), Institut Congolais pour la Conservation de la Nature (ICCN—unnumbered permit by Provincial Director of ICCN, Equateur Province in Mbandaka in August 2013, 004/ICCN/PNKB/2013, 06/ICCN/PNKB/2014, 02/ICCN/PNKB/2015), and Institut Superieur d’Ecologie Pour la Conservation de la Nature (ISEC, Katana—ISEC/DG/SGAC/04/2015, ISEC/DG/SGAC/04/29/2016). The University of Texas at El Paso (UTEP) Institutional Animal Care and Use Committee (IACUC—A-200902-1) approved field and laboratory methods. Permits for WC to collect snakes in South Africa were granted by the Department of Economic Development, Environmental Affairs and Tourism (permit nos. CRO 84/11CR and CRO 85/11CR). Permits for MOR and JP to collect snakes in Mozambique were granted by the Gorongosa Restoration Project and the Mozambican Departamento dos Serviços Cientificos (PNG/DSCi/C12/2013; PNG/DSCi/C12/2014; PNG/DSCi/C28/2015). Additional specimens and samples were obtained from natural history museums and university collections (Table 1) that followed appropriate legal guidelines and regulations for collection and loans of specimens.

**Table 1:** Voucher numbers, localities, and GenBank accession numbers for genetic samples. DRC = Democratic Republic of the Congo; RC = Republic of Congo; SA = South Africa; GNP = herpetological collection of the E. O. Wilson Biodiversity Center, Gorongosa National Park, Mozambique. Other collection acronyms are explained in Sabaj [108]. Note that Lawson et al. [109] erroneously listed the specimen of *Atractaspis* sp. as MVZ 228653.

Species	Collection No.	Field No.	Locality	*16S*	*ND4*	*cyt b*	*c-mos*	*RAG1*
*Eutropis longicaudata*	SAMA R38916	—	Malaysia	—	AY169645	DQ239139	DQ238979	—
*Rena humilis*	CAS 190589	—	—	—	—	—	—	—
*Boa constrictor*	—	—	—	—	—	AF471036	AF471115	—
*Acrochordus granulatus*	—	—	—	—	U49296	AF217841	AF471124	—
*Agkistrodon piscivorus*	—	—	—	—	AF156578	AF471074	AF471096	—
*Atheris nitschei*	—	—	—	—	AY223618	AF471070	AF471125	—
*Crotalus viridis*	—	—	—	—	AF194157	AF471066	AF471135	—
*Diadophis punctatus*	—	—	—	—	AF258910	AF471094	AF471122	—
*Hypsiglena torquata*	—	—	—	—	U49309	AF471038	AF471159	—
*Natrix natrix*	—	—	—	—	AY873710	AF471059	AF471121	—
*Thamnophis sirtalis*	—	—	—	—	AF420196	AF402929	DQ902094	—
*Boiga dendrophila*	—	—	—	—	U49303	AF471089	AF471128	—
*Bamanophis dorri*	—	—	—	—	AY487042	AY188040	AY188001	—
*Dolicophis jugularis*	—	—	—	—	AY487046	AY376740	AY376798	—
*Dendroaspis polylepis*	—	—	—	—	AY058974	AF217832	AY058928	—
*Naja kaouthia*	—	—	—	—	AY058982	AF217835	AY058938	—
*Naja annulata*	—	—	—	—	AY058970	AF217829	AY058925	—
*Bothrolycus ater*	—	—	—	—	—	—	—	—
*Gonionotophis brussauxi*	IRSNB 16266	—	Gabon: Ogooué-Lolo Province: Offoué-Onoy Department: Mount Iboundji	—	FJ404358	AY612043	AY611952	—
*Lycophidion capense*	PEM R22890	CMRK 275	Botswana	—	DQ486320	DQ486344	DQ486168	—
*Bothrophthalmus lineatus*	—	—	Uganda	—	—	AF471090	AF471090	—
*Lycodonomorphus laevissimus*	PEM R5630	—	SA: Eastern Cape Province: Grahamstown District	—	DQ486314	DQ486338	DQ486162	—
*Lycodonomorphus rufulus*	PEM R22892	CMRK 236	SA: Eastern Cape Province: Hole in the Wall	—	HQ207153	HQ207111	HQ207076	—
*Boaedon upembae*	UTEP 21002	ELI 205	DRC: Haut-Lomami Province: Kyolo	—	KM519681	KM519700	KM519734	KM519719
*Boaedon upembae*	UTEP 21003	ELI 208	DRC: Haut-Lomami Province: Kyolo	—	KM519680	KM519699	KM519733	KM519718
*Boaedon fuliginosus 1*	—	—	Burundi	—	FJ404364	FJ404302	AF544686	—
*Boaedon fuliginosus 2*	PEM R5639	—	Rwanda: Butare District	—	HQ207147	HQ207105	HQ207071	—
*Boaedon fuliginosus 3*	PEM R5635	—	Rwanda: Nyagatare District	—	HQ207148	HQ207106	HQ207072	—
*Psammophylax variabilis*	—	IPMB J296	Burundi	—	FJ404328	AY612046	AY611955	—
*Atractaspis andersonii*	MVZ 236612	—	Yemen: Lahi Governorate	—	—	MK621624	—	—
*Atractaspis andersonii*	MVZ 236613	—	Yemen: Lahi Governorate	MK621482	MK621565	MK621623	—	—
*Atractaspis andersonii*	MVZ 236614	—	Yemen: Lahi Governorate	—	—	MK621622	—	—
*Atractaspis* cf. *andersonii*	—	TMHC 2013-10-336	Oman: Dhofar Mts.	MK621475	MK621552	MK621609	—	—
*Atractaspis aterrima*	IRD CI.208	CI 208	Ivory Coast: Drekro	MK621477	MK621558	MK621615	MK621672	MK621521
*Atractaspis aterrima*	IRD CI.267	CI 267	Ivory Coast: Allakro	MK621478	MK621557	MK621614	MK621671	MG775793
*Atractaspis aterrima*	IRD T.265	TR 265	Togo: Mt. Agou	—	—	MK621616	MK621673	—
*Atractaspis aterrima*	—	TR 649	Mali	—	MK621559	MK621617	—	—
*Atractaspis bibronii*	MCZ-R 184426	AMB 8268	SA: Limpopo Province	MK621481	MK621544	MK621602	—	—
*Atractaspis bibronii*	MCZ-R 184500	AMB 8364	SA: Limpopo Province	—	MK621545	MK621603	MK621667	—
*Atractaspis bibronii*	MCZ-R 184505	AMB 8369	SA: Limpopo Province	—	MK621543	MK621601	—	MK621509
*Atractaspis bibronii*	PEM R20775	624	SA: Limpopo Province: Ngala	—	MK621534	MK621593	MK621663	—
*Atractaspis bibronii*	PEM R9768	629	Malawi: Mt. Mulanje	—	MK621535	MK621594	—	—
*Atractaspis bibronii*	PEM R20951	MB 21278	SA: Northern Cape Province: Kathu	—	MK621536	MK621595	—	MK621503
*Atractaspis bibronii*	—	MB 21703	SA: Mpumalanga Province: Madimola	MK621468	—	MK621598	MG775900	MG775791
*Atractaspis bibronii*	NMB R10815	MBUR 00961	SA: Limpopo Province: Tshipise region	MK621466	MK621537	MK621596	MK621664	MK621504
*Atractaspis bibronii*	NMB R10866	MBUR 20911	SA: Northern Cape Province: Boegoeberg Dam	—	MK621538	—	MK621665	MK621505
*Atractaspis bibronii*	—	MCZ-R 27182	SA: Limpopo Province	—	MK621546	MK621604	MK621668	—
*Atractaspis bibronii*	—	LV 004	SA: North West Province: Lephalale	—	MK621541	MK621599	MK621659	MK621510
*Atractaspis bibronii*	—	RSP 489	—	—	MK621540	—	—	—
*Atractaspis bibronii*	—	TGE-T2-36	SA: KwaZulu-Natal Province	MK621467	MK621539	MK621597	MK621666	MK621506
*Atractaspis bibronii rostrata*	—	GPN 191	Mozambique: Gorongosa National Park	MK621474	MK621542	MK621600	MK621660	MK621511
*Atractaspis bibronii rostrata*	—	GPN 353	Mozambique: Gorongosa National Park	MK621487	—	—	—	—
*Atractaspis bibronii rostrata*	—	GPN 354	Mozambique: Gorongosa National Park	MK621488	—	—	—	—
*Atractaspis bibronii rostrata*	—	GPN 421	Mozambique: Gorongosa National Park	MK621486	—	—	—	—
*Atractaspis bibronii rostrata*	—	MTSN 8354	Tanzania: Nguru Mts.	MK621490	—	—	—	—
*Atractaspis bibronii rostrata*	—	MTSN 8473	Tanzania: Usambara Mts.	MK621491	—	—	—	—
*Atractaspis bibronii rostrata*	MUSE 13889	—	Tanzania: Udzungwa Mts.	MK621489	—	—	—	—
*Atractaspis* cf. *bibronii rostrata*	UTEP 21661	ELI 038	DRC: Haut-Katanga Province: Pweto	MK621459	MK621532	MK621591	MK621661	MK621507
*Atractaspis* cf. *bibronii rostrata*	UTEP 21662	ELI 144	DRC: Haut-Katanga Province: Kabongo	MK621460	MK621533	MK621592	MK621662	MK621508
*Atractaspis boulengeri*	—	IPMB J355	Gabon: Ogooué-Maritime Province: Rabi	AY611833	FJ404334	AY612016	AY611925	—
*Atractaspis boulengeri*	—	29392	Gabon	MK621469	MK621551	MK621605	MK621658	MK621513
*Atractaspis boulengeri*	RBINS 18606	KG 063	DRC: Tshopo Province: Longala	—	MK621550	—	MK621657	MK621512
*Atractaspis boulengeri*	—	MSNS Rept 220	Gabon: Ivindo National Park: Ipassa	MK621493	—	—	—	—
*Atractaspis boulengeri*	IRSEN 00162	MBUR 03483	RC: Niari: Gnie-Gnie	MK621472	—	—	—	—
*Atractaspis congica*	—	633	Angola: Soyo	MK621461	MK621529	MK621587	MK621651	MG775788
*Atractaspis congica*	PEM R18087	CT 375	DRC	MK621462	—	MK621588	—	—
*Atractaspis congica*	PEM R22035	PVPL5 WRB	Angola: Luanda	—	MK621574	—	—	—
*Atractaspis corpulenta*	—	IPMB J369	Gabon: Ogooué-Maritime Province: Rabi	AY611837	FJ404335	AY612020	AY611929	—
*Atractaspis corpulenta*	PEM R22707	MBUR 03936	RC: Niari: Tsinguidi	MK621465	MK621548	MK621606	MK621654	MG775790
*Atractaspis corpulenta kivuensis*	RBINS 18607	CRT 4264	DRC: Tshopo Province: Lieki	—	MK621547	—	MK621655	—
*Atractaspis corpulenta kivuensis*	UTEP 21663	ELI 2992	DRC: Tshopo Province: Bombole	MK621471	MK621549	MK621607	MK621656	MK621514
*Atractaspis dahomeyensis*	IRD 2193.N	2193N Trape	Chad: Baibokoum	—	MK621561	MK621619	—	—
*Atractaspis dahomeyensis*	IRD 2197.N	2197N Trape	Chad: Baibokoum	MK621479	MK621560	MK621618	MK621674	—
*Atractaspis dahomeyensis*	IRD 5011.G	5011G Trape	Guinea: Kissidougou	MK621484	MK621562	—	—	—
*Atractaspis duerdeni*	—	MB 21346	SA: Northern Cape Province: Kuruman region	MK621463	MK621530	MK621589	MK621652	MG775789
*Atractaspis duerdeni*	—	MBUR 0229	SA: Limpopo Province: Senwabarwana region	MK621464	MK621531	MK621590	MK621653	MK621502
*Atractaspis* cf. *duerdeni*	—	—	Zimbabwe	—	U49314	AY188008	AY187969	—
*Atractaspis engaddensis*	TAUM 16030	—	Israel: Merav	—	MK621553	MK621610	—	—
*Atractaspis engaddensis*	TAUM 16542	—	Israel: Hare Gilboa	—	MK621554	MK621611	MG775901	MG775792
*Atractaspis engaddensis*	TAUM 17072	—	Israel: Yeroham	MK621476	MK621555	MK621612	MK621669	MK621519
*Atractaspis engaddensis*	TAUM 17094	—	Israel: Arad	—	MK621556	MK621613	MK621670	MK621520
*Atractaspis engaddensis*	—	3258WW	Saudi Arabia: Algassim	MG746902	—	—	—	—
*Atractaspis irregularis*	IRD 5010.G	5010G	Guinea: Kissidougou	—	MK621573	MK621625	—	—
*Atractaspis irregularis*	ZMB 87809	LI 10 104	Liberia: Nimba County	—	MK621568	MK621627	MK621646	MK621515
*Atractaspis irregularis*	ZMB 87867	LI 10 118	Liberia: Nimba County	—	MK621569	MK621628	MK621647	MK621516
*Atractaspis irregularis*	ZMB 88015	PLI 12 089	Liberia: Nimba County	MK621473	MK621570	MK621629	MK621648	MK621517
*Atractaspis irregularis*	IRD T.269	T 269	Togo: Mt. Agou	—	MK621566	—	MK621649	—
*Atractaspis irregularis*	IRD T.372	T 372	Togo: Diguengue	—	MK621567	—	MK621650	—
*Atractaspis* cf. *irregularis*	UTEP 21657	AKL 392	DRC: South Kivu Province: Lwiro	MK621492	—	—	—	—
*Atractaspis* cf. *irregularis*	UTEP 21658	EBG 1190	DRC: South Kivu Province: Lwiro	—	MG776014	MG746785	MG775898	—
*Atractaspis* cf. *irregularis*	UTEP 21659	EBG 2671	DRC: South Kivu Province: Lwiro	MK621457	MK621572	MK621631	MK621645	MK621518
*Atractaspis* cf. *irregularis*	UTEP 21660	EBG 2725	DRC: South Kivu Province: Lwiro	MK621458	—	—	—	—
*Atractaspis* cf. *irregularis*	UTEP 21654	ELI 1208	Burundi: Bubanza Province: Mpishi	MK621456	MK621571	MK621630	MK621644	MG775787
*Atractaspis* cf. *irregularis*	UTEP 21655	ELI 1635	DRC: South Kivu Province: Lwiro	MG746901	MG776015	—	MG775899	MG775786
*Atractaspis* cf. *irregularis*	MUSE 10470	—	DRC: South Kivu Province: Itombwe Plateau, Mulenge	MK621485	—	MK621626	—	—
*Atractaspis microlepidota*	No voucher	MBUR 08561	Ethiopia: Benishangul-Gumuz Province: Kutaworke region	MK621496	—	—	—	—
*Atractaspis microlepidota*	No voucher	MBUR 08365	Ethiopia: Benishangul-Gumuz Province: Kutaworke region	MK621494	—	—	—	—
*Atractaspis microlepidota*	No voucher	MBUR 08542	Ethiopia: Benishangul-Gumuz Province: Kutaworke region	MK621495	—	—	—	—
*Atractaspis micropholis*	IRD 1833.N	1833N Trape	Chad: Arninga Malick	MK621483	MK621575	—	—	—
*Atractaspis* cf. *micropholis*	—	IPMB J283	Togo	AY611823	FJ404336	AY612006	AY611915	—
*Atractaspis reticulata heterochilus*	UTEP 21664	ELI 2882	DRC: Tshopo Province: rd between Nia Nia and Kisangani	MK621470	MK621528	MK621586	—	—
*Atractaspis reticulata heterochilus*	UTEP 21665	ELI 3625	DRC: Maniema Province: Katopa, near Lomami National Park	—	—	MK621608	—	—
*Atractaspis reticulata heterochilus*	RBINS 18605	KG 219	DRC: Tshopo Province: Uma	—	MK621527	MK621585	MK621643	—
*Atractaspis reticulata heterochilus*	—	KG 495	DRC: Tshopo Province: Bagwase	—	MK621526	MK621584	MK621642	MK621501
*Atractaspis watsoni*	IRD 2523.N	2523N Trape	Chad: Balani	MK621480	MK621563	MK621620	MK621675	MK621522
*Atractaspis watsoni*	IRD 2565.N	2565N Trape	Chad: Balani	—	MK621564	MK621621	MK621676	MK621523
*Atractaspis* sp.	MVZ 229653	—	—	—	—	AF471046	AF471127	—
*Homoroselaps dorsalis*	PEM R:TBA	—	SA: Gauteng Province: Pretoria	MK621500	—	—	—	—
*Homoroselaps lacteus*	—	28676	SA: Gauteng Province: Pretoria	MK621497	—	MK621634	—	—
*Homoroselaps lacteus*	LSUMZ 57229	AMB 4483	SA: Eastern Cape Province: Port Elizabeth	MK621498	MK621581	MK621638	—	—
*Homoroselaps lacteus*	LSUMZ 55386	—	—	—	AY058976	—	AY058931	—
*Homoroselaps lacteus*	—	MCZ-R 28142	SA: Western Cape	—	MK621579	MK621636	—	—
*Homoroselaps lacteus*	—	MCZ-R 28271	SA: Western Cape: Mauritzbaai	—	MK621580	MK621637	—	—
*Homoroselaps lacteus*	PEM R17097	—	SA: Eastern Cape Province: Port Elizabeth	—	FJ404339	MK621635	FJ404241	—
*Homoroselaps lacteus*	PEM R17128	—	SA: Eastern Cape Province: Sundays River Mouth	—	MK621577	MK621633	—	MK621525
*Homoroselaps lacteus*	PEM R17129	—	SA: Eastern Cape Province: Sundays River Mouth	—	MK621576	MK621632	MK621677	MK621524
*Homoroselaps lacteus*	PEM R21097	WC 2688	SA: Eastern Cape Province: Thomas River	—	—	MK621640	—	—
*Homoroselaps lacteus*	PEM R19176	WC 10 092	SA: Free State Province: Reitz	MK621499	MK621583	MK621641	—	—
*Homoroselaps lacteus*	—	WC DNA 1261	SA: Mpumalanga Province: Wakkerstroom	—	MK621582	MK621639	—	—
*Amblyodipsas concolor*	—	634	SA: KwaZulu-Natal Province	—	MG775916	MG746801	MG775806	MG775720
*Amblyodipsas concolor*	PEM R17369	618	SA: KwaZulu-Natal Province: Cape Vidal	—	MG775917	MG746802	MG775807	MG775721
*Amblyodipsas concolor*	NMB R11375	MBUR 01624	SA: Limpopo Province: Wolkberg Wilderness Area	MG746916	MG775920	MG746804	MG775810	MG775724
*Amblyodipsas concolor*	NMB R11376	MBUR 01659	SA: Limpopo Province: Wolkberg Wilderness Area	—	MG775918	MG746803	MG775808	MG775722
*Amblyodipsas concolor*	NMB R11377	MBUR 01660	SA: Limpopo Province: Wolkberg Wilderness Area	MG746915	MG775919	—	MG775809	MG775723
*Amblyodipsas concolor*	PEM R19437	WC 373	SA: Eastern Cape Province: Hluleka	—	MG775922	MG746806	MG775812	MG775726
*Amblyodipsas concolor*	PEM R19795	WC 483	SA: Eastern Cape Province: Dwesa Point	—	MG775923	MG746807	MG775813	MG775727
*Amblyodipsas concolor*	PEM R20284	WC 975	SA: Eastern Cape Province: Mazeppa Bay	—	MG775921	MG746805	MG775811	MG775725
*Amblyodipsas dimidiata*	—	CMRK 311	Tanzania	—	DQ486322	DQ486346	DQ486170	—
*Amblyodipsas dimidiata*	PEM R15626	—	—	—	—	AY612027	AY611936	—
*Amblyodipsas microphthalma*	—	SP3	SA: Limpopo Province: Soutpansberg	MG746914	MG775927	MG746808	MG775818	MG775729
*Amblyodipsas polylepis*	—	AMB 6114	SA: Limpopo Province: Farm Guernsey	—	MG775932	—	MG775823	MG775734
*Amblyodipsas polylepis*	MCZ-R 190174	AMB 7960	Namibia: East Caprivi	—	MG775931	MG746812	MG775822	MG775733
*Amblyodipsas polylepis*	RBINS 18604	UP 052	DRC: Haut-Katanga Province: Kiubo	—	MG775929	MG746810	MG775820	MG775731
*Amblyodipsas polylepis*	PEM R22492	MBUR 00353	SA: Limpopo Province: Westphalia	MG746921	MG775928	MG746809	MG775819	MG775730
*Amblyodipsas polylepis*	PEM R18986	632	SA: Limpopo Province: Phalaborwa	—	MG775930	MG746811	MG775821	MG775732
*Amblyodipsas polylepis*	—	PVP9 WRB	Angola	MG746922	MG775933	MG746813	—	—
*Amblyodipsas polylepis*	—	MTSN 7571	Tanzania: Ruaha	MG746923	—	MG746814	—	—
*Amblyodipsas polylepis*	—	3128WW	—	MG746924	—	—	—	—
*Amblyodipsas polylepis*	PEM R23535	WC 4651	Angola: Moxico	MG746925	—	—	—	—
*Amblyodipsas unicolor*	—	PB-11-502	Guinea: Kankan	MG746917	MG775924	MG746815	MG775814	MG775728
*Amblyodipsas unicolor*	ZMB 88018	PGL-15-116	Ivory Coast: Yamassoukro	—	—	MG746816	MG775815	—
*Amblyodipsas unicolor*	IRD 2209.N	2209N Trape	Chad: Baibokoum	MG746918	MG775925	MG746817	MG775816	—
*Amblyodipsas unicolor*	IRD 2286.N	2286N Trape	Chad: Baibokoum	—	MG775926	MG746818	MG775817	—
*Amblyodipsas ventrimaculata*	PEM R23320	WC 3920	Angola: Moxico Province: Cuito River Source	MG746919	—	MG746819	—	—
*Amblyodipsas ventrimaculata*	—	R-SA	SA: Limpopo Province: Lephalale	MG746920	—	—	—	—
*Aparallactus capensis*	MCZ-R 184403	AMB 8180	SA: Eastern Cape Province: Farm Newstead	MG746971	MG776002	MG746888	MG775885	—
*Aparallactus capensis*	MCZ-R 184404	AMB 8181	SA: Eastern Cape Province: Farm Newstead	—	MG776003	MG746889	MG775886	—
*Aparallactus capensis*	MCZ-R 184501	AMB 8365	SA: Limpopo Province	—	MG776004	MG746890	MG775887	—
*Aparallactus capensis*	—	GPN 134	Mozambique: Gorongosa National Park	MG746988	MG776000	MG746886	MG775883	MG775781
*Aparallactus capensis*	ZMB 83259	GPN 310	Mozambique: Gorongosa National Park	MG746983	—	—	—	—
*Aparallactus capensis*	ZMB 83260	GPN 333	Mozambique: Gorongosa National Park	MG746979	—	—	—	—
*Aparallactus capensis*	—	GPN 351	Mozambique: Gorongosa National Park	MG746977	—	—	—	—
*Aparallactus capensis*	—	GPN 352	Mozambique: Gorongosa National Park	MG746978	—	—	—	—
*Aparallactus capensis*	ZMB 83342	GPN 359	Mozambique: Gorongosa National Park	MG746976	—	—	—	—
*Aparallactus capensis*	ZMB 83343	GPN 394	Mozambique: Gorongosa National Park	MG746981	—	—	—	—
*Aparallactus capensis*	ZMB 83261	GPN 429	Mozambique: Gorongosa National Park	MG746975	—	—	—	—
*Aparallactus capensis*	—	KB 2	Rwanda: Akagera National Park	—	MG775996	MG746882	MG775879	—
*Aparallactus capensis*	—	KB 5	Rwanda: Akagera National Park	MG746987	MG775995	MG746881	MG775878	MG775777
*Aparallactus capensis*	—	KB 8	Tanzania: Kigoma	—	MG775998	MG746884	MG775881	MG775779
*Aparallactus capensis*	—	KB 23	Rwanda: Akagera National Park	—	MG775997	MG746883	MG775880	MG775778
*Aparallactus capensis*	PEM R17909	648	Malawi: Mt. Mulanje	—	MG775984	MG746870	MG775867	MG775765
*Aparallactus capensis*	—	655	SA: Eastern Cape Province: Middleton	—	MG775987	—	MG775870	MG775768
*Aparallactus capensis*	PEM R17453	657	DRC: Lualaba Province: Kalakundi	MG746970	MG775986	—	MG775869	MG775767
*Aparallactus capensis*	PEM R17332	659	Tanzania: Klein’s Camp	—	MG775985	MG746871	MG775868	MG775766
*Aparallactus capensis*	HLMD J156	—	SA	AY188045	—	AY188006	AY187967	—
*Aparallactus capensis*	NMB R10885	MBUR 01229	SA: KwaZulu-Natal Province: Manyiseni	MG746985	—	MG746878	MG775876	—
*Aparallactus capensis*	NMB R11380	MBUR 01592	SA: Limpopo Province: Haenetsburg region	—	MG775992	MG746876	MG775875	MG775773
*Aparallactus capensis*	NMB R11381	MBUR 01593	SA: Limpopo Province: Haenetsburg region	—	MG775991	MG746875	MG775874	MG775772
*Aparallactus capensis*	NMB R11382	MBUR 01609	SA: Limpopo Province: Haenetsburg region	—	—	MG746873	MG775872	MG775770
*Aparallactus capensis*	NMB R11383	MBUR 01642	SA: Limpopo Province: Haenetsburg region	MG746984	MG775993	MG746877	—	MG775774
*Aparallactus capensis*	—	WC 1352	Mozambique: Cabo Delgado Province: Pemba	—	MG775999	MG746885	MG775882	MG775780
*Aparallactus capensis*	PEM R20693	WC 2612	SA: Eastern Cape Province: Tsolwana	—	MG775994	MG746880	MG775877	MG775776
*Aparallactus capensis*	—	MCZ-R 27164	SA: Limpopo Province	MG746973	—	MG746892	—	—
*Aparallactus* cf. *capensis*	PEM R18438	677	SA: Limpopo Province	—	MG775988	MG746872	MG775871	MG775769
*Aparallactus* cf. *capensis*	NMB R10997	MBUR 00871	SA: Limpopo Province: Cleveland Nature Reserve	MG746986	—	MG746879	—	MG775775
*Aparallactus* cf. *capensis*	NMB R11379	MBUR 01554	SA: Limpopo Province: near Sentrum	—	—	MG746874	MG775873	MG775771
*Aparallactus* cf. *capensis*	—	MCZ-R 27805	SA: Limpopo Province	MG746972	MG776005	MG746891	—	—
*Aparallactus* cf. *capensis*	—	GPN 242	Mozambique: Gorongosa National Park	MG746989	MG776001	MG746887	MG775884	MG775782
*Aparallactus* cf. *capensis*	—	GPN 357	Mozambique: Gorongosa National Park	MG746982	—	—	—	—
*Aparallactus* cf. *capensis*	ZMB 83344	GPN 403	Mozambique: Gorongosa National Park	MG746980	—	—	—	—
*Aparallactus* cf. *capensis*	—	2118 WW	SA: Limpopo Province: Bela Bela	MG746969	—	—	—	—
*Aparallactus* cf. *capensis*	—	2119 WW	SA: Limpopo Province: Bela Bela	MG746968	—	—	—	—
*Aparallactus* cf. *guentheri*	—	MTSN 8341	Tanzania: Nguru Mts	MG746974	—	MG746899	—	—
*Aparallactus* cf. *guentheri*	PEM R5678	—	Tanzania: Usambara Mts	—	—	AY235730	—	—
*Aparallactus jacksonii*	PEM R20739	649	Tanzania: Mt. Kilimanjaro	MG746960	MG775980	MG746866	—	—
*Aparallactus jacksonii*	PEM R17876	650	Tanzania: Oldonyo Sambu	MG746962	MG775983	MG746869	MG775866	MG775764
*Aparallactus jacksonii*	PEM R17874	651	Tanzania: Oldonyo Sambu	MG746961	MG775981	MG746867	MG775864	MG775762
*Aparallactus jacksonii*	PEM R17875	654	Tanzania: Ndukusiki	—	MG775982	MG746868	MG775865	MG775763
*Aparallactus jacksonii*	—	MTSN 8301	Tanzania: Nguru Mts	MG746963	—	—	—	—
*Aparallactus jacksonii*	—	MTSN 8303	Tanzania: Nguru Mts	MG746967	—	—	—	—
*Aparallactus jacksonii*	—	MTSN 8323	Tanzania: Nguru Mts	MG746964	—	—	—	—
*Aparallactus jacksonii*	—	MTSN 8352	Tanzania: Nguru Mts	MG746965	—	—	—	—
*Aparallactus jacksonii*	—	MTSN 8353	Tanzania: Nguru Mts	MG746966	—	—	—	—
*Aparallactus lunulatus*	—	653	Tanzania: Nguru Mts	MG746991	MG776006	—	MG775891	MG775784
*Aparallactus lunulatus*	IRD 2158.N	2158N	Chad: Baibokoum	—	MG776009	MG746896	MG775888	—
*Aparallactus lunulatus*	IRD 2178.N	2178N	Chad: Baibokoum	MG746993	MG776010	MG746897	MG775889	—
*Aparallactus lunulatus*	TMHC 2013-09-315	—	Ethiopia: Borana	MG746992	MG776008	MG746895	—	—
*Aparallactus lunulatus*	TMHC 2013-09-316	—	Ethiopia: Simien Mts.	—	MG776007	MG746894	—	—
*Aparallactus lunulatus*	—	WBR 957	NE of Lake Albert	MG746990	—	MG746893	MG775890	MG775783
*Aparallactus modestus*	—	IPMB J284	Gabon: Ogooué-Maritime Province: Rabi	AY611824	FJ404332	AY612007	AY611916	—
*Aparallactus modestus*	MCZ-R 182624	—	RC: Bomassa	—	—	MG746863	MG775862	—
*Aparallactus modestus*	MCZ-R 182625	—	RC: Bomassa	—	MG775977	MG746864	MG775863	—
*Aparallactus modestus*	MVZ 252411	—	Ghana: Ajenjua Bepo	MG746957	MG775978	MG746865	—	—
*Aparallactus modestus*	USNM 584365	—	RC: Impongui	MG746949	MG775958	MG746844	MG775844	MG775747
*Aparallactus modestus*	ZFMK 87627	—	—	MG746959	—	—	—	—
*Aparallactus modestus*	IRD 5009.G	5009G Trape	Guinea: Kissidougou	MG746958	MG775979	—	—	—
*Aparallactus modestus*	RBINS 18608	CRT 4045	DRC: Tshopo Province: Bomane	—	MG775964	MG746850	MG775850	—
*Aparallactus modestus*	—	CRT 4181	DRC: Tshopo Province: Lieki	—	MG775966	MG746852	—	MG775752
*Aparallactus modestus*	—	CRT 4256	DRC: Tshopo Province: Lieki	—	MG775967	—	—	MG775753
*Aparallactus modestus*	UTEP 21609	EBG 2609	DRC: Ituri Province: Bazinga	MG746950	MG775959	MG746845	MG775845	—
*Aparallactus modestus*	UTEP 21605	ELI 1379	DRC: South Kivu Province: Kihungwe	MG746951	MG775960	MG746846	MG775846	MG775748
*Aparallactus modestus*	UTEP 21606	ELI 1419	DRC: South Kivu Province: Kihungwe	MG746952	MG775961	MG746847	MG775847	MG775749
*Aparallactus modestus*	No voucher	ELI 2138	DRC: Equateur Province: Npenda Village	MG746948	MG775957	MG746843	—	—
*Aparallactus modestus*	UTEP 21601	ELI 2221	DRC: Equateur Province: Npenda Village	MG746953	MG775962	MG746848	MG775848	—
*Aparallactus modestus*	UTEP 21602	ELI 2222	DRC: Equateur Province: Npenda Village	MG746954	MG775963	MG746849	MG775849	MG775750
*Aparallactus modestus*	UTEP 21608	ELI 2914	DRC: Tshopo Province: Kisangani	MG746955	MG775968	MG746853	MG775852	—
*Aparallactus modestus*	—	KG 457	DRC: Tshopo Province: Bagwase	—	MG775970	MG746855	MG775855	MG775755
*Aparallactus modestus*	—	KG 467	DRC: Tshopo Province: Bagwase	—	MG775972	MG746858	MG775858	MG775758
*Aparallactus modestus*	—	KG 499	DRC: Tshopo Province: Bagwase	—	MG775973	—	MG775859	MG775759
*Aparallactus modestus*	—	KG 501	DRC: Tshopo Province: Bagwase	—	MG775971	MG746857	MG775857	MG775757
*Aparallactus modestus*	—	KG 503	DRC: Tshopo Province: Bagwase	—	MG775969	MG746854	MG775854	MG775754
*Aparallactus modestus*	—	KG 511	DRC: Tshopo Province: Bagwase	—	MG775975	MG746860	MG775861	MG775761
*Aparallactus modestus*	—	KG 528	DRC: Tshopo Province, Bagwase	—	—	MG746856	MG775856	MG775756
*Aparallactus modestus*	—	KG 572	DRC: Tshopo Province: Bagwase	—	MG775974	MG746859	MG775860	MG775760
*Aparallactus modestus*	—	MSNS REPT 34	Gabon: Ogooué-Lolo Province: Mt. Iboundji	—	—	MG746862	—	—
*Aparallactus modestus*	—	PB 11-733	Guinea: Nzerekore	—	MG775976	MG746861	MG775853	—
*Aparallactus modestus*	RBINS 18603	UAC 038	DRC: Tshopo Province: Yoko	—	MG775965	MG746851	MG775851	MG775751
*Aparallactus modestus*	PEM R22331	MBUR 03449	RC: Niari: Doumani	MG746956	—	—	—	—
*Aparallactus niger*	IRD 8075.X	8075X	Guinea: Nzerekore	MG746994	MG776011	MG746898	MG775892	—
*Aparallactus werneri*	FMNH 2504400	—	Tanzania: Tanga	—	U49315	AF471035	—	—
*Chilorhinophis gerardi*	PEM R18882	635	Zambia: Kalumbila	MG746995	MG776012	MG746900	MG775893	MG775785
*Macrelaps microlepidotus*	PEM R20944	—	SA: KwaZulu-Natal Province: Hillcrest	MG746927	MG775938	—	—	—
*Macrelaps microlepidotus*	—	28666	—	—	MG775935	MG746821	MG775824	—
*Macrelaps microlepidotus*	PEM R19791	WC DNA 511	SA: Eastern Cape Province: Dwessa Nature Reserve	MG746926	MG775934	MG746820	—	—
*Macrelaps microlepidotus*	PEM R20167	WC DNA 928	SA: Eastern Cape Province: Hogsback	—	MG775937	MG746823	—	—
*Macrelaps microlepidotus*	PEM R20295	WC DNA 973	SA: Eastern Cape Province: Mazeppa Bay	—	MG775936	MG746822	—	—
*Micrelaps bicoloratus*	—	CMRK 330	—	—	—	*DQ486349*	*DQ486173*	—
*Micrelaps muelleri*	TAUM 15654	—	Israel: Salti	—	—	MG746781	—	—
*Micrelaps muelleri*	TAUM 16469	—	Israel: Malkishua	—	—	MG746782	MG775895	—
*Micrelaps muelleri*	TAUM 16738	—	Israel: Bet Nehemya	—	—	MG746783	MG775896	—
*Micrelaps muelleri*	TAUM 16944	—	Israel: Ein Hod	—	MG776013	MG746784	MG775897	—
*Micrelaps* cf. *muelleri*	TAUM 16426	—	Israel: Afiq	—	—	MG746780	MG775894	—
*Polemon acanthias*	—	PEM R1479	Ivory Coast: Haute Dodo	AY611848	FJ404341	AY612031	AY611940	—
*Polemon acanthias*	ZMB 88016	PLI-12-053	Liberia: Nimba County	—	MG775954	MG746841	MG775841	MG775745
*Polemon acanthias*	ZMB 88017	PLI-12-208	Liberia: Nimba County	MG746946	MG775955	MG746842	MG775842	MG775746
*Polemon acanthias*	IRD T.266	T266 Trape	Togo: Mt. Agou	MG746947	MG775956	—	MG775843	—
*Polemon ater*	PEM R17452	—	DRC: Lualaba Province: Kalakundi	MG746943	MG775951	MG746838	MG775839	MG775743
*Polemon ater*	PEM R20734	—	DRC: Lualaba Province: Fungurume	MG746944	MG775952	MG746839	MG775840	MG775744
*Polemon christyi*	UTEP 21618	DFH 535	Uganda: Western Region: road to Budongo Central Forest Reserve	MG746945	MG775953	MG746840	—	—
*Polemon collaris*	PEM R19893	TB 28	Angola: North-west region	MG746931	MG775943	MG746827	MG775829	—
*Polemon collaris*	UTEP 21612	ELI 561	DRC: South Kivu Province: vicinity of Byonga	MG746928	MG775939	MG746824	MG775825	MG775735
*Polemon collaris*	UTEP 21613	ELI 1317	DRC: South Kivu Province: Fizi	MG746930	MG775941	MG746826	MG775827	MG775737
*Polemon collaris*	UTEP 21614	ELI 2464	DRC: Tshuapa Province: Watsi Kengo, Salonga River	MG746929	MG775940	MG746825	MG775826	MG775736
*Polemon collaris*	—	KG 523	DRC: Tshopo Province: Bagwase	—	MG775944	MG746828	MG775830	—
*Polemon collaris*	—	MSNS REPT 110	Gabon: Ogooué-Lolo Province: Mt. Iboundji	MG746934	—	MG746829	—	—
*Polemon collaris*	RBINS 18544	UAC 62	DRC: Tshopo Province: Yoko	MG746933	MG775942	—	MG775828	—
*Polemon collaris*	PEM R22747	MBUR 03862	RC: Niari: Tsinguidi region	MG746932	—	—	—	—
*Polemon fulvicollis*	PEM R5388		Gabon: Ogooué-Maritime Province: Rabi	AY611846	FJ404342	AY612029	AY611938	—
*Polemon fulvicollis laurenti*	UTEP 21615	ELI 3046	DRC: Tshopo Province: Bombole Village	MG746942	MG775949	MG746837	MG775837	—
*Polemon graueri*	RBINS 18543	CRT 4007	DRC: Tshopo Province: Bomane	—	MG775947	MG746833	MG775834	MG775740
*Polemon graueri*	UTEP 21610	EBG 1376	DRC: South Kivu Province: Irangi	MG746940	—	MG746835	MG775836	MG775742
*Polemon graueri*	No voucher	EBG 2294	DRC: Ituri Province: Komanda	MG746938	—	MG746832	MG775833	—
*Polemon graueri*	UTEP 21611	ELI 2842	Uganda: Western Region: Rwenzori Mts National Park	MG746939	MG775948	MG746834	MG775835	MG775741
*Polemon graueri*	—	MTSN 7378	Rwanda: Nyungwe National Park	MG746941	—	MG746836	—	—
*Polemon notatus*	—	29395	Gabon	MG746935	MG775950	—	MG775838	—
*Polemon notatus*	PEM R5404	—	Gabon: Ogooué-Maritime Province: Rabi	AY611847	FJ404343	AY612030	AY611939	—
*Polemon* cf. *robustus*	UTEP 21617	ELI 2594	DRC: Equateur Province: Salonga River	MG746936	MG775945	MG746830	MG775831	MG775738
*Polemon robustus*	UTEP 21616	ELI 2069	DRC: Mai-Ndombe Province: Isongo, Lake Mai-Ndombe	MG746937	MG775946	MG746831	MG775832	MG775739
*Xenocalamus bicolor*	—	MCZ-R 27160	SA: Limpopo Province	—	MG775911	MG746794	MG775800	—
*Xenocalamus bicolor*	—	MCZ-R 27161	SA: Limpopo Province	MG746905	MG775912	MG746795	MG775801	—
*Xenocalamus bicolor*	PEM R17377	615	SA: Northern Cape Province: Kimberly	—	MG775903	—	MG775795	MG775710
*Xenocalamus bicolor*	PEM R17438	616	SA: KwaZulu-Natal Province	—	—	MG746787	—	—
*Xenocalamus bicolor*	PEM R17438	647	SA: Northern Cape Province: Kimberly, Rooipoort	—	MG775902	MG746786	MG775794	MG775709
*Xenocalamus bicolor*	NMB R10851	MBUR 00925	SA: Limpopo Province: Woudend	MG746904	MG775910	MG746793	MG775799	MG775716
*Xenocalamus bicolor*	NMB R11418	MBUR 01553	SA: Limpopo Province: Sentrum	—	MG775907	MG746790	MG775797	MG775714
*Xenocalamus bicolor*	—	TGE T3 28	SA: Northern Cape Province	—	MG775905	MG746788	MG775796	MG775712
*Xenocalamus bicolor*	—	TGE T3 29	SA: Northern Cape Province	—	MG775908	MG746791	MG775798	MG775715
*Xenocalamus bicolor*	—	TGE T3 32	SA: Northern Cape Province	—	MG775909	MG746792	—	—
*Xenocalamus bicolor*	—	TGE T4 14	SA: Free State Province	—	MG775906	MG746789	—	MG775713
*Xenocalamus bicolor australis*	PEM R22083	—	SA: Northern Cape Province: Kimberly	MG746906	MG775913	MG746796	MG775802	—
*Xenocalamus bicolor lineatus*	—	13321	—	—	—	MG746797	MG775803	—
*Xenocalamus bicolor machadoi*	PEM R20771	666	Angola: Moxico	MG746903	MG775904	—	—	MG775711
*Xenocalamus mechowii*	PEM R23533	WC 4654	Angola: Moxico	MG746908	—	—	—	—
*Xenocalamus mechowii*	PEM R23463	WC 4695	Angola: Cuando Cubango	MG746907	—	—	—	—
*Xenocalamus michelli*	UTEP 21619	ELI 209	DRC: Haut-Lomami Province: Kyolo	MG746909	MG775914	MG746798	MG775804	MG775718
*Xenocalamus michelli*	UTEP 21620	ELI 355	DRC: Tanganyika Province: near Manono airport	MG746910	MG775915	MG746799	MG775805	MG775719
*Xenocalamus transvaalensis*	NMB R10888	MBUR 01107	SA: KwaZulu-Natal Province: Ndumo Game Reserve	MG746913	—	MG746800	—	MG775717
*Xenocalamus transvaalensis*	—	FO57-51-51	SA: KwaZulu-Natal Province: Maputaland	MG746911	—	—	—	—
*Xenocalamus transvaalensis*	PEM R:TBA	—	SA: KwaZulu-Natal Province: Hluhluwe	MG746912	—	—	—	—
*Xenocalamus transvaalensis*	PEM R12103	—	SA: KwaZulu-Natal Province: Maputaland	AY611842	FJ404344	AY612025	AY61193	—

### 2.2 Taxon sampling

**Fig 1:**
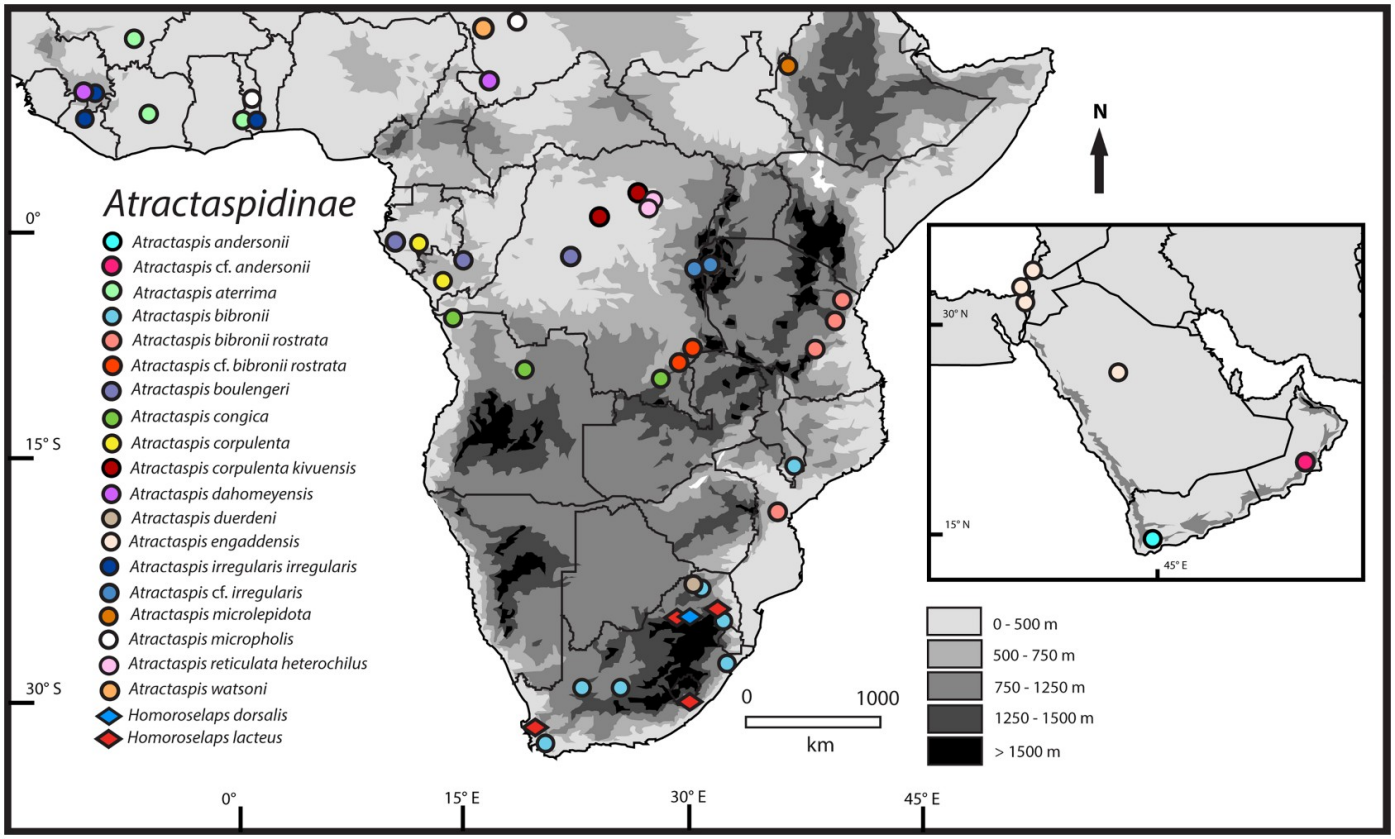
Map of sub-Saharan Africa and western Asia/Middle East, showing sampling localities for atractaspidines used in this study.

**Fig 2:**
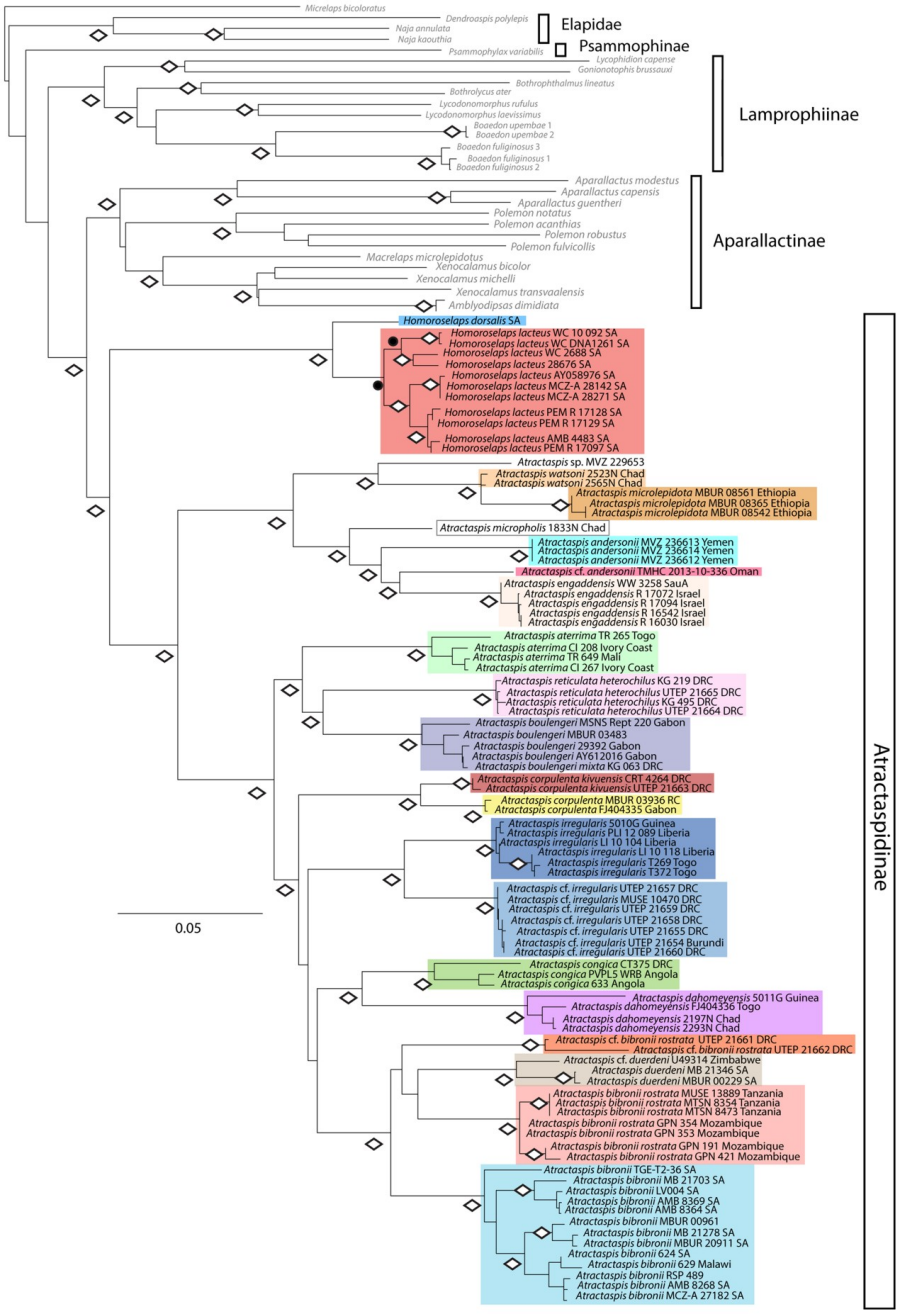
Maximum-likelihood phylogeny of Atractaspidinae with combined 16S, ND4, cyt *b*, c-mos, and RAG1 data sets. Closed circles denote clades with Bayesian posterior probability values ≥ 0.95. Diamonds denote clades with strong support in both maximum likelihood analyses (values ≥ 70) and Bayesian analyses (posterior probability values ≥ 0.95).

Specimens from the Subfamily Atractaspidinae were collected from multiple localities in sub-Saharan Africa (Fig 1). We generated sequences of three mitochondrial genes (*16S*, *ND4*, and *cyt b*) and two nuclear genes (*c-mos* and *RAG1*) for 91 atractaspidine individuals (Tables 1 and 2). This study included sequences from both atractaspidine genera (14/22 species of *Atractaspis*; 2/2 species of *Homoroselaps*) [24, 34]. Sequences from some of these individuals have been published previously [2, 7], and new sequences were deposited in GenBank (Table 1). Concatenated trees were rooted with *Acrochordus granulatus* (not shown on Fig 2). Three genera of Viperidae (*Agkistrodon*, *Atheris*, and *Crotalus*; not shown on Fig 2), two genera of Elapidae (*Naja* and *Dendroaspis*), six genera of Lamprophiinae (*Boaedon*, *Bothrophthalmus*, *Bothrolycus*, *Gonionotophis*, *Lycodonomorphus*, and *Lycophidion*), *Psammophylax*, and *Micrelaps* were used as outgroups for the concatenated analyses (Table 1, Fig 2). Additionally, we included sequences from six of the eight known aparallactine genera (6/9 species of *Amblyodipsas*; 7/11 species of *Aparallactus*; 1/2 species of *Chilorhinophis*; 1/1 species of *Macrelaps*; 7/14 species of *Polemon*; 4/5 species of *Xenocalamus*) [24, 35] for concatenated analyses and ancestral-state reconstructions. For divergence-dating analyses, additional samples from the squamate taxa Scincidae, Leptotyphlopidae, Viperidae, Colubrinae, and Dipsadinae were included (Table 1).

**Table 2:** Primers used for sequencing mitochondrial and nuclear genes.

Gene Name	Primer Name	Primer Sequence ('5 to 3')	Primer Source
**16S**	L2510	CGCCTGTTTATCAAAAACAT	[110]
	H3059	CCGGTCTGAACTCAGATCACGT	
	L2510mod/16Sar	CCGACTGTTTAMCAAAAACA	[111]
	H3056mod/16Sbr	CTCCGGTCTGAACTCAGATCACGTRGG	
**ND4**	ND4	CACCTATGACTACCAAAAGCTCATGTAGAAGC	[64, 112]
	HIS1276	TTCTATCACTTGGATTTGCACCA	
**cyt *b***	L14910	GACCTGTGATMTGAAAAACCAYCGTTGT	[109, 113]
	H16064	CTTTGG TTTACAAGAACAATGCTTTA	
**c-mos**	S77	CATGGACTGGGATCAGTTATG	[114]
	S78	CCTTGGGTGTGATTTTCTCACCT	
**RAG1**	G396 (R13)	TCTGAATGGAAATTCAAGCTGTT	[115]
	G397 (R18)	GATGCTGCCTCGGTCGGCCACCTTT	

### 2.3 Laboratory protocols

Genomic DNA was isolated from alcohol-preserved muscle or liver tissue samples with the Qiagen DNeasy tissue kit (Qiagen Inc., Valencia, CA, USA). Primers used herein are shown in Table 2. We used 25 μL PCR reactions with gene-specific primers with an initial denaturation step of 95°C for 2 min, followed by denaturation at 95°C for 35 seconds (s), annealing at 50°C for 35 s, and extension at 72°C for 95 s with 4 s added to the extension per cycle for 32 (mitochondrial genes) or 34 (nuclear gene) cycles. Amplification products were visualized on a 1.5% agarose gel stained with SYBR Safe DNA gel stain (Invitrogen Corporation, Carlsbad, CA, USA). Sequencing reactions were purified with CleanSeq magnetic bead solution (Agencourt Bioscience, La Jolla, CA) and sequenced with an ABI 3130xl automated sequencer at the University of Texas at El Paso (UTEP) Genomic Analysis Core Facility.

### 2.4 Sequence alignment and phylogenetic analyses

Phylogenetic analyses were conducted for our individual and five-gene concatenated data sets. Data were interpreted using the program SeqMan [36]. An initial alignment for each gene was produced in MUSCLE [37] in the program Mesquite v3.10 [38], and manual adjustments were made in MacClade v4.08 [39]. The Maximum Likelihood (ML) analyses of single gene and concatenated data sets were conducted using the GTRGAMMA model in RAxML v8.2.9 via the Cipres Science Gateway v3.3 [40]. All parameters were estimated, and a random starting tree was used. Support values for clades inferred by ML analyses were assessed with the rapid bootstrap algorithm with 1,000 replicates [40]. We also conducted Bayesian inference (BI) analyses with MrBayes v3.2.6 via the Cipres Science Gateway [40]. The model included 13 data partitions: independent partitions for each codon position of the protein-coding genes *ND4*, *cyt b*, *c-mos*, and *RAG1*, and a single partition for the mitochondrial gene *16S*. Phylogenies were constructed based on concatenated data, which included *16S* and the four protein-coding genes listed above. Concatenated data sets were partitioned identically for ML and BI analyses. The program PartitionFinder v1.1.1 [41–42] was used to find the model of evolution that was most consistent with our data for BI analyses. Bayesian analyses were conducted with random starting trees, run for 20,000,000 generations, and sampled every 1000 generations. Phylogenies were visualized using FigTree v1.3.1 [43].

### 2.5 Divergence dating

The program BEAST v1.8.3 via Cipres Science Gateway [40] was used to estimate divergence times across atractaspidine phylogenetic estimates. The five-gene data set was used to estimate divergence dates in BEAST. Substitution and clock models were unlinked for all partitions; trees were unlinked across the nuclear loci, but were linked for the two mitochondrial partitions because these evolve as a single unit. We implemented an uncorrelated log-normal relaxed clock model with a Yule tree prior. Two independent analyses were run for 100 million generations, sampling every 10,000 generations. Primary calibration points were obtained from Head et al. [44] and a secondary calibration point was obtained from Kelly et al. [7] including: the split between Scolecophidia and all other snakes (120–92 mya); split between Caenophidia and its nearest sister taxon, Booidea (72.1–66 mya); split between Colubroidea and its nearest sister taxon (*Acrochordus* + Xenodermatidae) (72.1–50.5 mya); the divergence of Colubridae + Elapoidea (30.9 ± 0.1 mya); and the split between Crotalinae and Viperinae (23.8–20.0 mya). All calibrations were constrained with a log-normal mean of 0.01, a normal standard deviation of 2.0 (first calibration point), and 1.0 (the last four calibration points). Parameter values of the samples from the posterior probabilities on the maximum clade credibility tree were summarized using the program TreeAnnotator v1.8.3 via Cipres Science Gateway [40].

### 2.6 Ancestral-state reconstructions

To understand the evolution of fang morphology and diet selection in atractaspidines, we reconstructed the pattern of character changes on the ML phylogeny herein. For ancestral-state reconstructions, we included all samples of aparallactines and atractaspidines available to us in order to better characterize fang and diet characters. All ancestral-state reconstructions were conducted by tracing characters over trees in Mesquite v3.10 [38]. We scored taxa using descriptions from the literature [25, 30–31, 45–55], and from our own data. We evaluated the following characters for fang morphology and diet selection: A. Fang morphology: (0) no fang, (1) rear fang, (2) fixed front fang, (3) moveable front fang, and (4) rear-front fang intermediate (anterior half of the maxilla, but not the anteriormost tooth); B. prey selection (0) rodents, (1) rodents, snakes, fossorial lizards, and amphibians, (2) snakes, (3) amphisbaenians, (4) snakes and fossorial lizards, (5) invertebrates, and (6) fish and amphibians. A ML approach was used for both analyses, because it accounts for and estimates probabilities of all possible character states at each node, thus providing an estimate of uncertainty [56]. A Markov K-state one-parameter model (Mk-1; [57]) that considers all changes as equally probable was implemented in our ancestral-state reconstructions. States were assigned to nodes if their probabilities exceeded a decision threshold; otherwise nodes were recovered as equivocal.

### 2.7 Morphology

Microcomputed tomography (CT) scans of specimens were produced using GE Phoenix V|Tome|X systems at the General Electric Sensing & Inspection Technologies in Scan Carlos, CA and University of Florida’s Nanoscale Research Facility. X-ray tube voltage and current, detector capture time, voxel resolution, and projection number were optimized for each specimen (S1 File). The radiographs were converted into tomograms with Phoenix Datos| R, and then rendered in three dimensions with volumetric rendering suite VGStudioMax 3.2 (http://www.volumegraphics.com). Tomogram stacks and 3D mesh files for all scans are available on Morphosource.org (S1 File).

## 3. Results

### 3.1 Concatenated gene tree analyses

Our data set consisted of 3933 base pairs (*16S* [546 bp], *ND4* [679 bp], *cyt b* [1094 bp], *c-mos* [605 bp], and *RAG1* [1009 bp]). Individuals with missing data were included in the concatenated sequence analyses, because placement of individuals that are missing a significant amount of sequence data can be inferred in a phylogeny, given an appropriate amount of informative characters [8, 58–60]. Furthermore, Jiang et al. [61] showed that excluding genes with missing data often decreases accuracy relative to including those same genes, and they found no evidence that missing data consistently bias branch length estimates.

The following models of nucleotide substitution were selected by PartitionFinder for BI analyses: *16S* (GTR+G), *ND4* 1^st^ codon position (GTR+G), *ND4* 2^nd^ codon position (TVM+G), and *ND4* 3^rd^ codon position (HKY+I+G); *cyt b* 1^st^ codon position (TVM+G), *cyt b* 2^nd^ codon position (HKY+I+G) and *cyt b* 3^rd^ codon position (GTR+G); *c-mos* and *RAG1* 1^st^, 2^nd^ and 3^rd^ codon positions (HKY+I). Preferred topologies for the ML and BI analyses were identical, with similar, strong support values for most clades (Fig 2), and single-gene mtDNA analyses recovered similar topologies (not shown). The ML analysis likelihood score was –46340.867388. The relationships of Elapidae, Lamprophiinae, *Micrelaps*, and *Psammophylax* with respect to the ingroup Atractaspidinae, were not strongly supported in ML and BI analyses. However, Atractaspidinae was recovered in a strongly supported clade. *Atractaspis* and *Homoroselaps* were strongly supported as sister taxa (Fig 2). The genus *Homoroselaps* was recovered as a monophyletic group, and *H*. *lacteus* was partitioned into several well-supported clades. There were several strongly supported clades within *Atractaspis*: (1) *Atractaspis andersonii*, (2) *Atractaspis aterrima*, (3) *A*. *bibronii*, (4) *A*. *bibronii rostrata*, (5) *A*. cf. *bibronii rostrata*, (6) *A*. *boulengeri*, (7) *A*. *congica*, (8) *A*. *corpulenta corpulenta*, (9) *A*. *corpulenta kivuensis*, (10) *A*. *dahomeyensis*, (11) *A*. *duerdeni*, (12) *A*. *engaddensis*, (13) *A*. *irregularis*, (14) *A*. cf. *irregularis*, (15) *A*. *reticulata heterochilus*, and (16) *A*. *microlepidota*. There was strong support for a western Asia/Middle East and Africa clade containing *A*. *andersonii*, *A*. *engaddensis*, *A*. *microlepidota*, *A*. *micropholis*, *A*. *watsoni*, and *A*. sp. *Atractaspis andersonii* did not form a monophyletic group, because one of the samples from Oman (AF471127) was recovered as sister to a clade of *A*. *engaddensis* with strong support (Fig 2). The western African species *A*. *aterrima* was recovered with strong support as sister to a clade containing *A*. *reticulata heterochilus* and *A*. *boulengeri*. *Atractaspis corpulenta kivuensis* samples from eastern DRC were strongly supported as sister to *A*. *corpulenta* from northwestern Republic of Congo (near Gabon, the type locality). A well-supported clade of *Atractaspis irregularis* samples was partitioned by strongly supported central (*A*. cf. *irregularis*) and western African (*A*. *irregularis*) subclades. *Atractaspis duerdeni* was recovered within a well-supported *A*. *bibronii* complex. *Atractaspis bibronii rostrata* samples were partitioned into two highly divergent clades from southeastern DRC and Tanzania/Mozambique.

**Fig 3:**
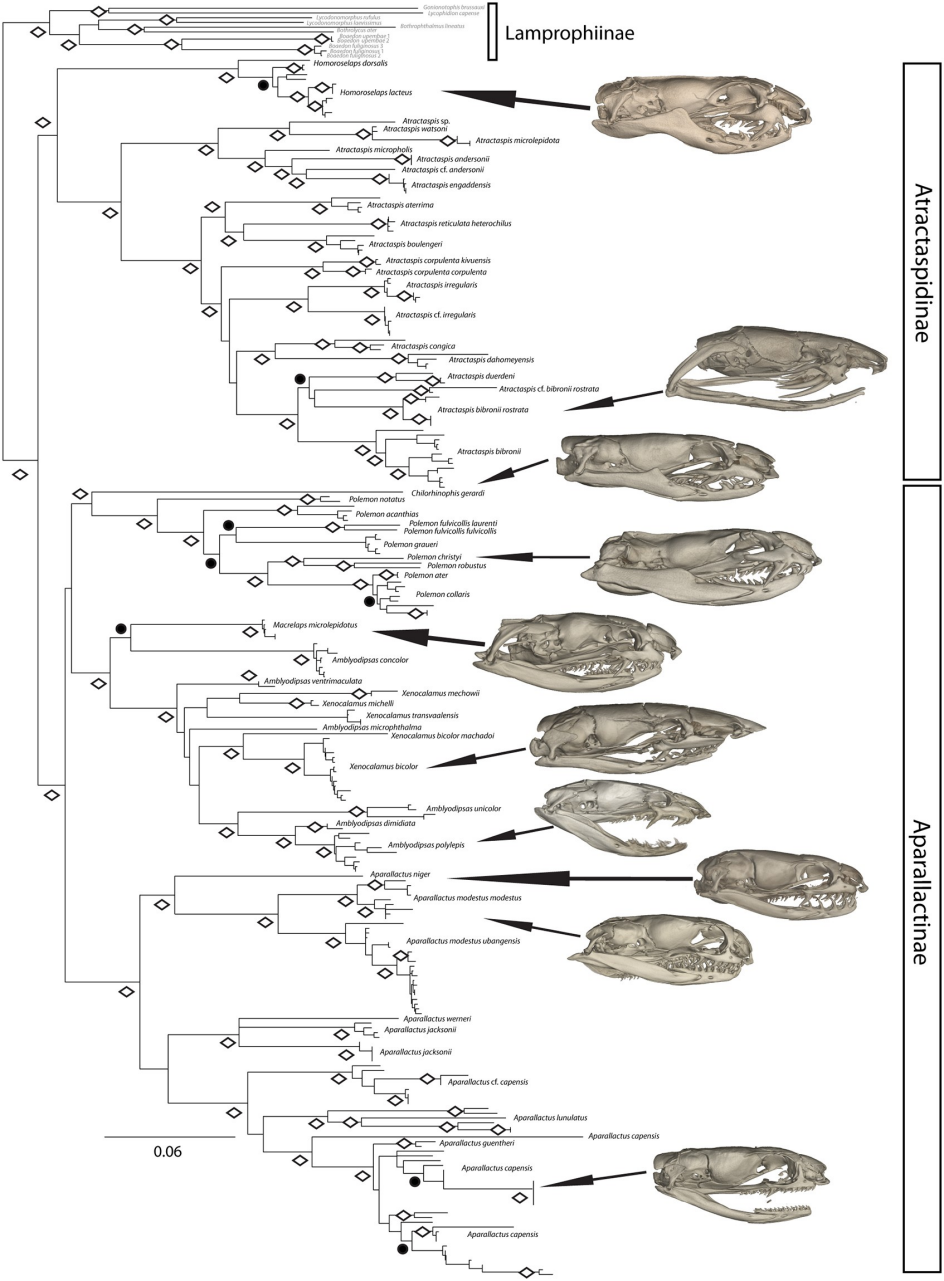
Maximum-likelihood phylogeny of Atractaspidinae and Aparallactinae with combined 16S, ND4, cyt *b*, c-mos, and RAG1 data sets. Diamonds denote clades with maximum likelihood values ≥ 70 and Bayesian posterior probability values ≥ 0.95; closed circles denote clades with Bayesian posterior probability values ≥ 0.95.

For the analyses including all atractaspidine and aparallactine samples available to us (Fig 3), preferred topologies for the ML and BI analyses were identical, with similar, strong support values for most clades (Fig 3). The ML analysis likelihood score was –73090.650849. The concatenated ML and BI analyses recovered similar topologies to those from Portillo et al. [62] and Fig 2.

### 3.2 Divergence dating

**Fig 4:**
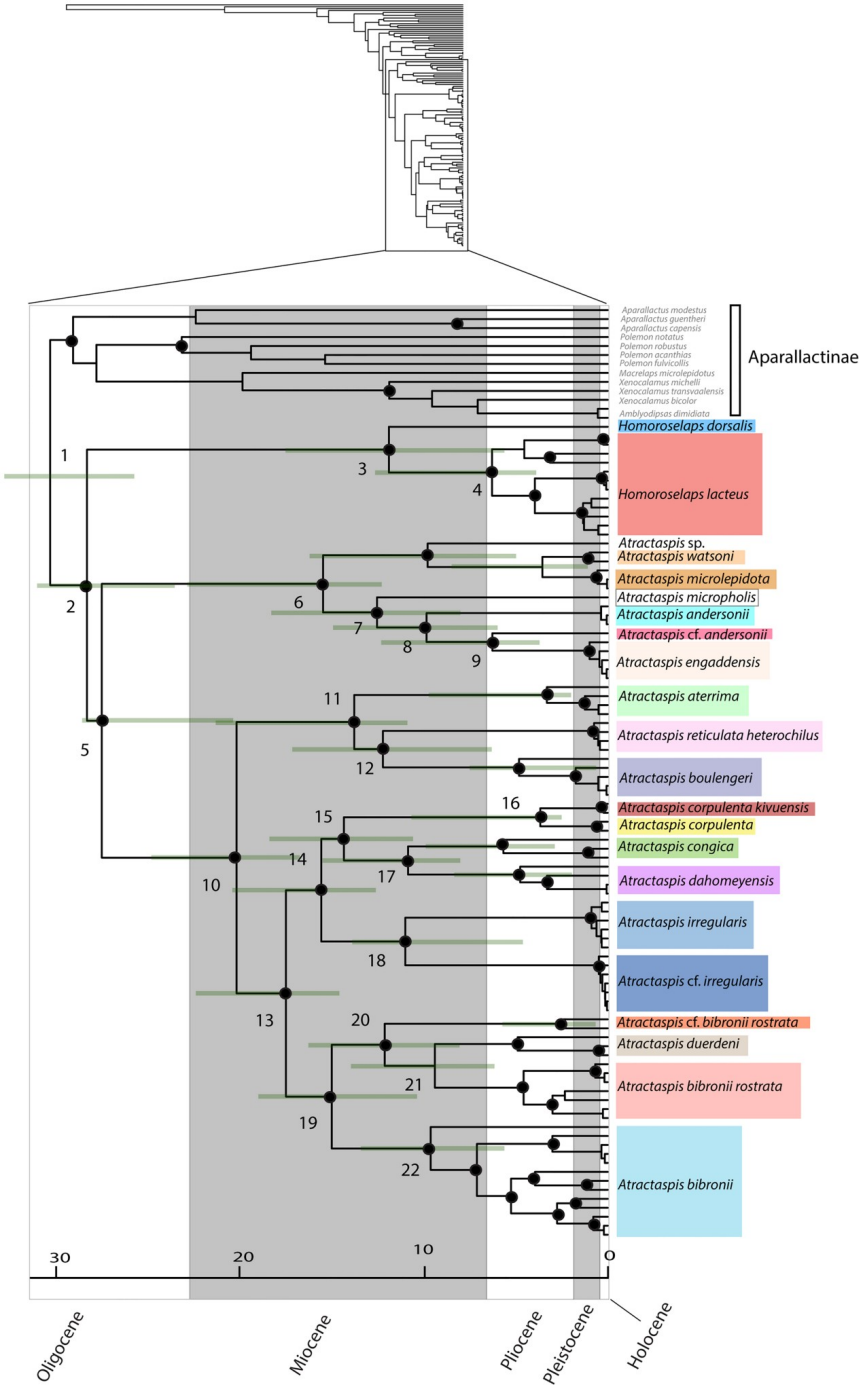
Phylogeny resulting from BEAST, based on four calibration points. Nodes with high support (posterior probability ≥ 0.95) are denoted by black circles. Median age estimates are provided along with error bars representing the 95% highest posterior densities (HPD) (Table 3).

Topologies from the BEAST (Fig 4) analyses were mostly consistent with the results from our concatenated tree analyses (Figs 2 and 3). BEAST results recovered *A*. *corpulenta corpulenta*/*A*. *corpulenta kivuensis* as sister to *A*. *congica*/*A*. *dahomeyensis* with strong support (Figs 2–4). Additionally, the relationship between *Atractaspis irregularis* and *A*. *corpulenta*/*A*. *congica*/*A*. *dahomeyensis* was strongly supported in BEAST analyses (Fig 4). Results from dating analyses suggested atractaspidines split from aparallactines during the early Oligocene around 29 mya (24.8–31.4 mya, 95% highest posterior densities [HPD]) (Table 3, Fig 4), which is similar to the results (34 mya) of Portillo et al. [62]. Subsequently, *Atractaspis* split from *Homoroselaps* in the mid-Oligocene, and most radiation events within each of the major clades associated with these genera occurred during the mid- to late Miocene and Pliocene (Fig 4). Specific dates with ranges are specified in Table 3.

**Table 3:** Estimated dates and 95% highest posterior densities (HPD) of main nodes. Node labels correspond to those in Fig 4.

Node	Event	Estimated age in mya (95% HPD)
**1**	Split between Aparallactinae and Atractaspidinae	29.1 (24.8–31.4)
**2**	Split between *Homoroselaps* and *Atractaspis*	27.2 (22.5–29.7)
**3**	Split between *Homoroselaps dorsalis* and *H*. *lacteus*	11.4 (5.3–16.8)
**4**	Basal divergence of *Homoroselaps lacteus*	6.0 (3.6–12.2)
**5**	Basal divergence of *Atractaspis*	26.4 (19.6–27.4)
**6**	Split between *A*. *watsoni*/*A*. *microlepidota*/*A*. sp. and *A*. *micropholis*/*A*. *andersonii*/*A*. cf. *andersonii/A*. *engaddensis*	14.8 (11.7–21.9)
**7**	Split between *A*. *micropholis* and *A*. cf. *andersonii*/*A*. *engaddensis*/*A*. *andersonii*	12.1 (7.8–17.6)
**8**	Split between *A*. cf. *andersonii*/*A*. *engaddensis* and *A*. *andersonii*	9.5 (5.7–14.4)
**9**	Split between *A*. cf. *andersonii* and *A*. *engaddensis*	6.0 (3.6–11.7)
**10**	Split between *A*. *aterrima*/*A*. *boulengeri*/*A*. *reticulata* and the remainder of *Atractaspis*	19.4 (16.1–23.7)
**11**	Split between *A*. *aterrima* and *A*. *boulengeri*/*A*. *reticulata*	13.2 (10.5–20.4)
**12**	Split between *A*. *boulengeri* and *A*. *reticulata*	11.7 (6.1–16.5)
**13**	Split between *A*. *corpulenta*/*A*. *congica*/*A*. *dahomeyensis*/*A*. *irregularis* and *A*. *duerdeni*/*A*. *bibronii* complex	16.8 (14.1–21.5)
**14**	Split between *A*. *corpulenta*/*A*. *congica*/*A*. *dahomeyensis* and *A*. *irregularis*	14.9 (12.1–19.6)
**15**	Split between *A*. *corpulenta* and *A*. *dahomeyensis*/*A*. *congica*	13.8 (10.2–17.6)
**16**	Split between *A*. *corpulenta corpulenta* and *A*. *corpulenta kivuensis*	3.6 (2.5–10.2)
**17**	Split between *A*. *congica* and *A*. *dahomeyensis*	10.4 (7.6–14.8)
**18**	Split between *A*. *irregularis irregularis* and *A*. cf. *irregularis*	10.5 (4.4–13.2)
**19**	Basal divergence of the *A*. *bibronii* complex	14.4 (10.1–18.3)
**20**	Split between *A*. cf. *bibronii rostrata* and *A*. *duerdeni*/*A*. *bibronii rostrata*	11.6 (7.6–15.7)
**21**	Split between *A*. *bibronii rostrata* and *A*. *duerdeni*	9.0 (5.8–13.4)
**22**	Basal divergence of *A*. *bibronii*	9.2 (5.6–12.9)

### 3.3 Ancestral-state reconstructions

**Fig 5:**
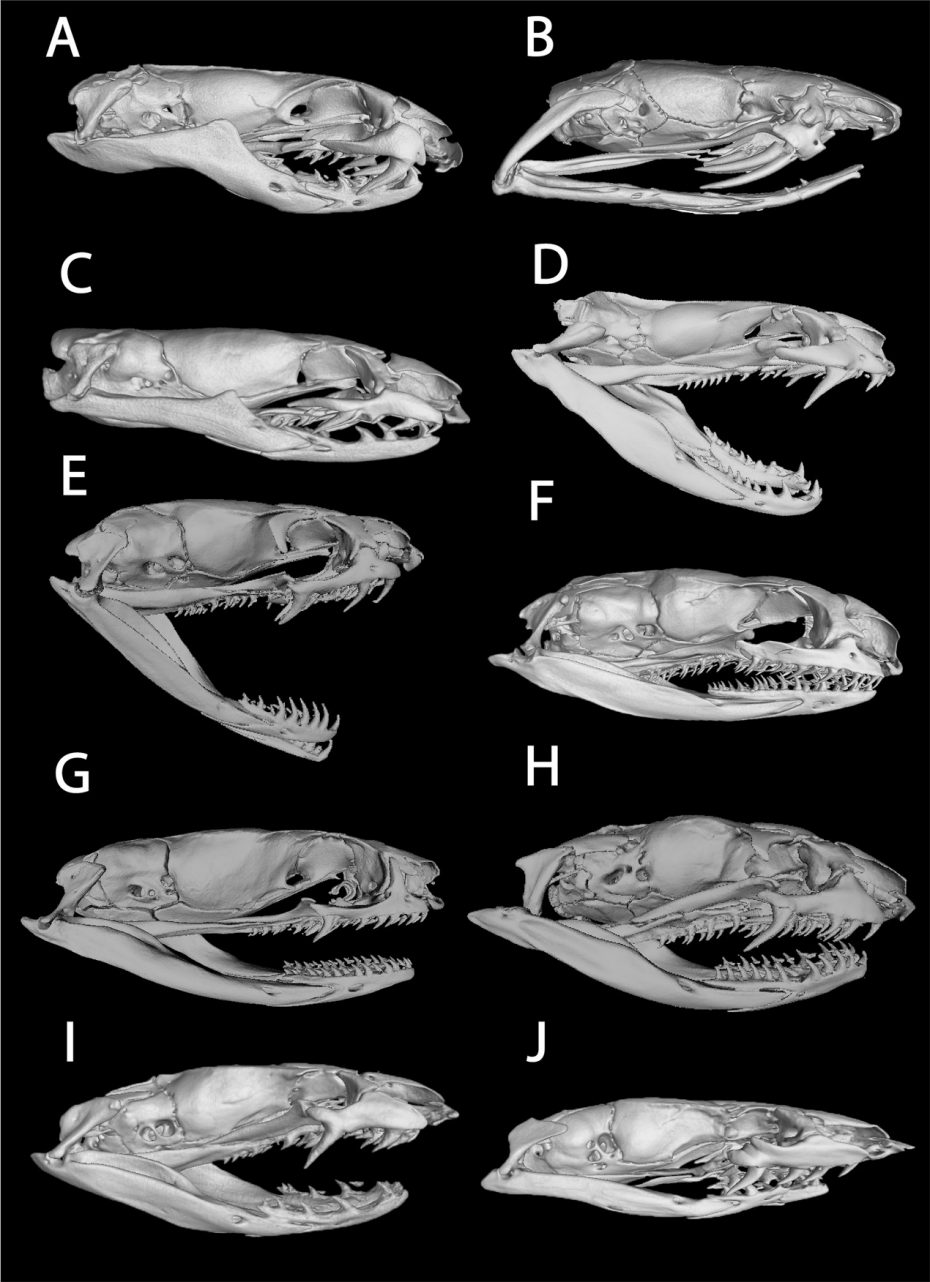
Computed tomography (CT) scans of aparallactine and atractaspidine genera. *Homoroselaps lacteus* (CAS 173258) (A); *Atractaspis bibronii* (CAS 111670) (B); *Chilorhinophis gerardi* (CAS 159106) (C); *Polemon christyi* (CAS 147905) (D); *Aparallactus niger* (AMNH 142406) (E); *Aparallactus modestus* (CAS 111865) (F); *Aparallactus capensis* (G); *Macrelaps microlepidotus* (H); *Amblyodipsas polylepis* (CAS 173555) (I); *Xenocalamus bicolor* (CAS 248601) (J).

**Fig 6:**
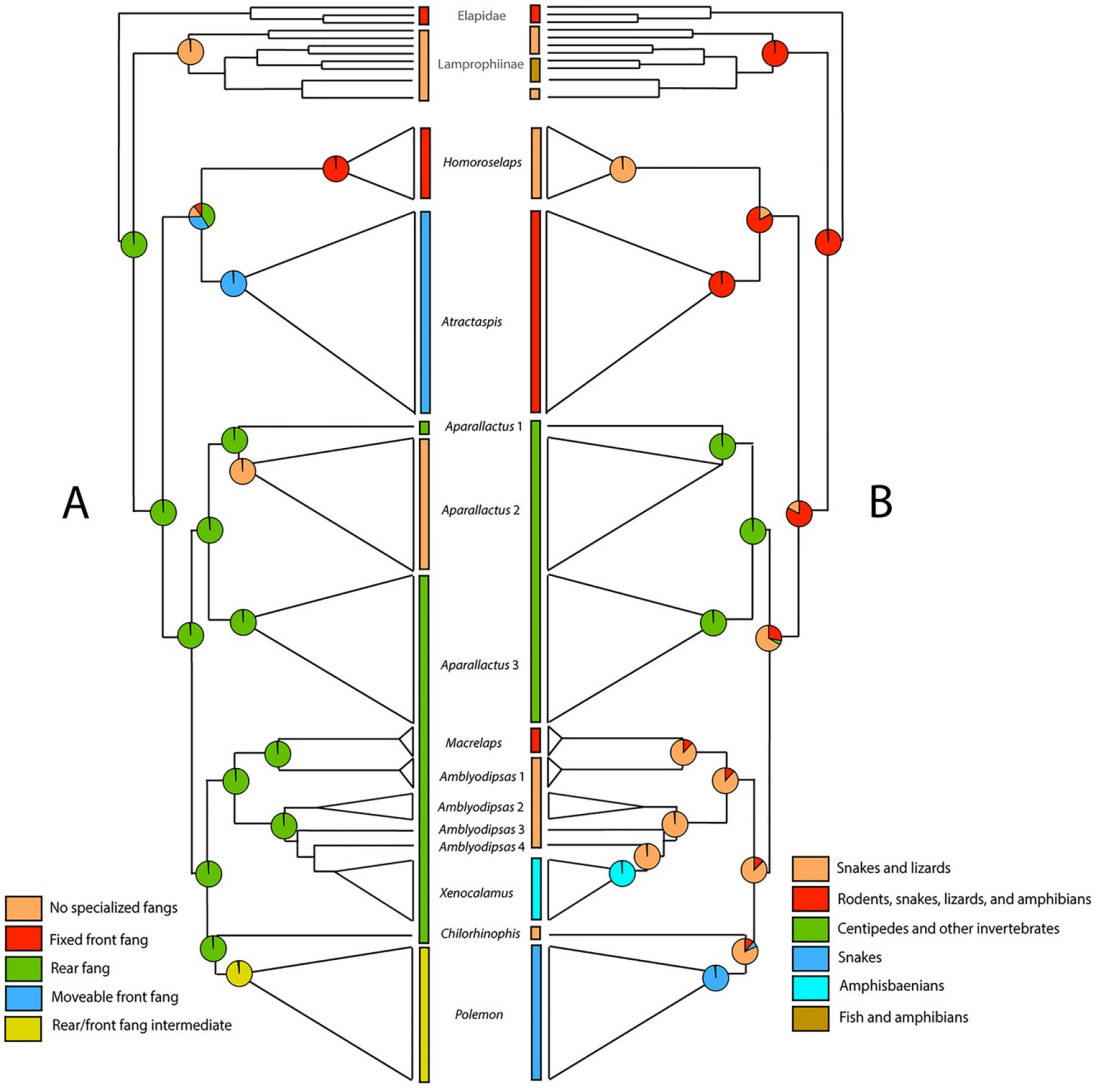
Ancestral-state reconstructions with ML optimization on the ML trees from the concatenated analyses shown in Fig 2. (A) fang morphology, (B) dietary preference. *Aparallactus* 1 = *A*. *niger*; *Aparallactus* 2 = *A*. *modestus*; *Aparallactus* 3 = *A*. *capensis*, *A*. cf. *capensis*, *A*. *guentheri*, *A*. *jacksonii*, *A*. *lunulatus*, and *A*. *werneri*; *Amblyodipsas* 1 = *A*. *concolor*; *Amblyodipsas* 2 = *A*. *dimidiata*, *A*. *polylepis*, and *A*. *unicolor*; *Amblyodipsas* 3 = *A*. *ventrimaculata*; *Amblyodipsas* 4 = *A*. *microphthalma*.

X-ray computer tomography of collared snakes and burrowing asps can be seen in Figs 3 and 5. Likelihood reconstructions of atractaspidine ancestral fang morphology inferred a rear fang condition for the ancestral condition of all lamprophiids (96.7%) (Fig 6[A]). Subsequently, the Subfamily Lamprophiinae lost a venom delivery fang condition. The common ancestor of aparallactines and atractaspidines was inferred to have a rear fang condition (97.8%). The analyses suggested a rear fang ancestor (72.5%) for the clade containing *Homoroselaps* and *Atractaspis*. The ancestor to *Atractaspis* was inferred to have a moveable front fang condition (97.4%). Results recovered a fixed front fang condition for the ancestor of all *Homoroselaps* (99.8%). The ancestor to all aparallactines was inferred to have a rear fang condition (99.6%), and this remained consistent throughout most aparallactine nodes with the exception of *Polemon* (rear/front fang intermediate, 97.8%) and *Aparallactus modestus* (no specialized fang, 99.7%).

For the analyses with diet data, likelihood reconstructions inferred a generalist diet of rodents, reptiles, and amphibians for the ancestral condition of all lamprophiids (99.7%) (Fig 6[B]). Several lamprophiines (*Lycodonomorphus*) subsequently adopted a more specialized diet of amphibians, reptiles, and fish. The common ancestor for aparallactines and atractaspidines was inferred to have a generalist diet of rodents, reptiles, and amphibians (92.4%). Results recovered a more specialized ancestral diet of snakes and lizards (64.5%) for aparallactines, which was favored over a generalist diet (27.7%). The condition of a snake and lizard diet (79.9%) was favored over a generalist diet (16.2%) for the ancestor of *Polemon*/*Chilorhinophis* and *Amblyodipsas*/*Macrelaps*/*Xenocalamus*. The latter dietary condition was retained for the ancestor of *Polemon*/*Chilorhinophis* (79.4%) and the ancestor of *Amblyodipsas*/*Macrelaps*/*Xenocalamus* (87.6%). Specialized dietary conditions were recovered for the genera *Aparallactus* (centipedes and other invertebrates, 99.7%), *Polemon* (snakes, 97.8%), and *Xenocalamus* (amphisbaenians, 98.8%). Results suggested a generalist diet for Atractaspidinae (92.3%). The ancestor of *Homoroselaps* was inferred to have a diet consisting of mostly lizards and snakes (99.9%), whereas the ancestor of *Atractaspis* was inferred to have a broader diet of rodents, reptiles, and amphibians (99.2%).

## 4. Discussion

### 4.1 Biogeography

Atractaspidines are distributed throughout sub-Saharan Africa except for three species of *Atractaspis* that are found in western Asia/Middle East (*Atractaspis andersonii*, *A*. *engaddensis*, and *A*. *microlepidota*) [25, 29–31]. Based on our results, the most likely scenario for *Atractaspis* is an African origin with a vicariance or dispersal event into the western Asia/Middle East region in the late Miocene (Fig 4). *Atractaspis* from western Asia/Middle East and Africa last shared a common ancestor during the late Miocene around 12.1 mya (7.8–17.6). Other studies of African-western Asian/Middle Eastern complexes (e.g., *Echis* and *Uromastyx*) recovered similar dates during the late Miocene, with the Red Sea proving to be a strong biogeographic barrier [63–69]. However, lineages of *Varanus* from Africa and the Middle East split from each other 6.9 mya [70], and African and Middle Eastern *Bitis arietans* last shared a common ancestor around 4 mya [64]. These dating estimates suggest that there were multiple dispersal events, which were taxon specific. Many Middle Eastern amphibians and reptiles have common ancestors in the Horn of Africa [63–71]. Our study lacked multiple *Atractaspis* species from the Horn of Africa, and future studies should include samples of *A*. *fallax*, *A*. *magrettii*, *A*. *leucomelas*, and *A*. *scorteccii* to improve understanding of likely Africa–Asia biogeographic patterns in atractaspidines.

*Atractaspis* began to diversify around the mid-Oligocene simultaneously with many aparallactine genera [62]. Many of the modern species split from recent common ancestors during the mid- to late Miocene (Table 3, Fig 4). The late Miocene was characterized by considerable xeric conditions, which led to the expansion of savannas globally [72–73]. Other studies on Central and East African herpetofauna, including squamates (*Adolfus*, *Atheris*, *Boaedon*, *Naja*, *Kinyongia*, and *Panaspis*) and frogs (*Amietia*, *Leptopelis*, and *Ptychadena*), have shown similar trends of species diversification during the late Miocene [3–5, 62, 74–78].

The diversification of several western and central African *Atractaspis* was most likely a consequence of increasingly xeric conditions during the Miocene, when forest and other moist habitats were fragmented [72]. These *Atractaspis* were likely isolated in fragmented patches of forest during the mid- to late Miocene. *Atractaspis irregularis* is partitioned clearly by western African and central African lineages that diverged in the mid-Miocene, similar to *Aparallactus modestus* [62]. At this time, southern African and Middle Eastern *Atractaspis* also diversified. *Atractaspis* from the Near and Middle East (*A*. *andersonii*, *A*. *engaddensis*, and *A*. *microlepidota*) and southern Africa (*A*. *bibronii* and *A*. *duerdeni*) are not tropical forest species, and they inhabit deserts or semi-desert savannas and dry woodland [30, 79–80]. This adaptation to more xeric and open habitats would have allowed Near and Middle Eastern, and southern African *Atractaspis*, to disperse into these habitats during the dry conditions of the mid- to late Miocene. Studies on mammals and birds show most diversification events during the Pliocene [81–84], which is consistent with the timing of diversification for *Atractaspis aterrima*, *A*. *congica*, *A*. *dahomeyensis*, and populations of South African *A*. *bibronii* (Fig 4).

In contrast to *Aparallactus jacksonii*, *Atractaspis bibronii rostrata* showed no clear genetic partitioning between populations in the Nguru, Usambara, and Udzungwa Mountains [62]. *Aparallactus jacksonii* clearly exhibited deep divergence between an extreme northern Tanzanian population, and a population from the Nguru Mountains. These two populations diverged from each other during the late Miocene, suggesting that the habitats of this taxon were fragmented with increased aridity [62]. Other vertebrate taxa that have shown substantial divergences between populations found in extreme northern Tanzania (Usambara, Taita, and Pare Mountains) and those slightly south (Uluguru, Ukaguru, Nguru, and Malundwe Mountains), include the reed frog *Hyperolius puncticulatus*, the green barbet (*Stactolaema olivacea*), and the streaky canary (*Serinus striolatus*) [82, 85]. But like *Atractaspis bibronii rostrata*, the hyperoliid reed frog *Hyperolius spinigularis* and the aparallactine *Aparallactus guentheri* showed no clear biogeographic patterns between populations in different areas of the Eastern Arc Mountains. These results support the hypothesis that the evolutionary history of species from the Eastern Arc Mountains is lineage specific [85]. *Atractaspis bibronii rostrata* inhabit low-elevation woodlands and grasslands, and transitional habitats, rather than montane forest (i.e., *Aparallactus jacksonii*) [25]. This would allow taxa such as *Atractaspis bibronii rostrata* to continuously disperse between the different mountains of the Eastern Arcs, despite increased aridity. Additionally, ecological niche requirements may also explain the different biogeographic patterns seen in *Aparallactus jacksonii* and *Atractaspis bibronii rostrata*. *Atractaspis bibronii* has a generalist diet (mammals, squamates, and amphibians) and could have exploited more habitats than *Aparallactus jacksonii*, which is a centipede specialist [25].

### 4.2 Evolutionary relationships and taxonomy of Atractaspidinae

Our results indicate that both *Atractaspis* and *Homoroselaps* are strongly supported as monophyletic sister taxa. Results from Figueroa et al. [27] recovered a monophyletic group containing aparallactines and atractaspidines, but their results did not recover a monophyletic *Atractaspis* (*A*. *irregularis* was recovered as sister to aparallactines + atractaspidines). This sample was excluded from our analyses, because the only sequence available for this taxon was from *BDNF*, a gene not used herein. The results from Figueroa et al. [27] may be an artifact of sample size of atractaspidines, or incomplete lineage sorting of the *BDNF* nuclear gene. Results from our study indicate that *A*. *irregularis* is a monophyletic lineage within a strongly supported, monophyletic *Atractaspis*.

Underwood and Kochva [18] recognized two groups within *Atractaspis*: (1) the ‘*bibronii*’ group (represented in our study by *A*. *aterrima*, *A*. *bibronii*, *A*. *boulengeri*, *A*. *congica*, *A*. *corpulenta*, *A*. *dahomeyensis*, *A*. *irregularis*, and *A*. *reticulata*), characterized by a single posterior supralabial, three anterior infralabials, normal-sized venom glands, and a sub-Saharan distribution; and (2) the ‘*microlepidota*’ group (represented in our study by *A*. *andersonii*, *A*. *engaddensis*, *A*. *microlepidota*, and *A*. *micropholis*), characterized by two anterior temporals, highly elongated venom glands, and a North African/Near and Middle Eastern distribution. Whereas our study did not include genetic samples of all known species of *Atractaspis*, results herein (Fig 2) support partitioning of the genus into two groups *sensu* Underwood and Kochva [18]. Our results indicated a clear partition between a ‘Middle Eastern + African’ clade (including *A*. *watsoni*, a species that was not included by Underwood and Kochva [18]) and a ‘sub-Saharan African’ clade (Figs 2 and 4). These results strengthen the notion that venom gland size and length in *Atractaspis* are homologous. Our support for the ‘*microlepidota*’ group is consistent with the “Section A” (*A*. *andersonii*, *A*. *fallax*, *A*. *leucomelas*, *A*. *microlepidota*, and *A*. *micropholis*) of Laurent [28] and the *A*. *micropholis*/*A*. *microlepidota*/*A*. *watsoni* clade recovered by Moyer and Jackson [10]. However, our phylogeny (Fig 2) contrasts with the remaining “sections” of Laurent [28], most relationships depicted in the morphological phylogeny of Moyer and Jackson [10], and the molecular phylogenies of Pyron et al. [8–9] and Vidal et al. [22].

Based on relatively long branch lengths, several lineages of *Atractaspis* seem to be cryptic complexes of species. Because of the extensive geographic distribution of *A*. *bibronii* in central, eastern and southern Africa, it is unsurprising to find several highly divergent lineages that likely represent cryptic species. Given the proximity (ca. 167–333 km) of our Tanzanian localities of *A*. *bibronii rostrata* (Nguru, Usambara, and Udzungwa Mountains) to the insular type locality for this taxon (Zanzibar, Tanzania), the morphological similarity between our voucher specimens and the types [86], and the relatively long branch length and reciprocal monophyly of this clade compared to topotypic South African *A*. *bibronii* (Fig 2), it is likely that the former taxon is a valid species. However, additional comparisons to type specimens are needed to clarify the taxonomic status of populations in this clade, including samples from Haut-Katanga Province in southeastern DRC.

Our phylogenetic results indicated that several other species, including *A*. *andersonii*, *A*. *boulengeri*, *A*. *congica*, *A*. *corpulenta*, *A*. *dahomeyensis*, and *A*. *irregularis* likely represent more than a single species. For example, topotypic Angolan samples of *A*. *congica* are deeply divergent from our eastern DRC sample (Fig 2), which is likely attributable to *A*. *congica orientalis* [46]. Like *Polemon fulvicollis fulvicollis* (Gabon) and *P*. *fulvicollis laurenti* (DRC) [62], Gabonese *Atractaspis corpulenta* and eastern DRC populations of *A*. *corpulenta kivuensis* also showed marked genetic divergences between each other (Fig 2). The well-supported clade of *A*. *irregularis* from western Africa likely includes topotypic populations, because they straddle the type locality (Accra, Ghana) [87], whereas our Albertine Rift samples are likely attributable to one of the taxon’s many synonyms. One of these, *Atractaspis bipostocularis* from Mt. Kenya, was named for its two postocular scales, which distinguishes it from the single postocular of topotypic *A*. *irregularis* [88]. Because Mt. Kenya is located east of the Kenyan Rift, a major biogeographic barrier to several species of squamates [78], and moreover, all voucher specimens of *A*. cf. *irregularis* from the Albertine Rift have a single postocular (EG pers. obs.), *A*. *bipostocularis* is likely a distinct species that is endemic to the central Kenyan highlands. Other synonyms of *A*. *irregularis* that have one postocular and type localities in or near the Albertine Rift are likely attributable to our well-supported clade of *A*. cf. *irregularis* (Fig 2 in [87]), and include the following taxa: *A*. *conradsi* Sternfeld, 1908 (type locality: Ukerewe Island, Lake Victoria, Tanzania [89]), *A*. *schoutedeni* de Witte, 1930 (type locality: Goma, North Kivu, DRC [90]), *A*. *babaulti* Angel, 1934 (type locality: Kadjuju [1500 m elevation] on the western border of Lake Kivu, 15 km north of Katana, DRC [91]), and *A*. *irregularis loveridgei* Laurent, 1945 (type locality: Bunia, DRC [46]). Additional sampling and morphological analyses are in progress that will help clarify the correct taxonomy for these lineages. Because of the relative lack of fieldwork in Central Africa in recent decades [92–93] and the relatively rare encounters of these snakes above ground (EG, pers. obs.), it is likely that genetic samples from the above topotypic populations will remain elusive for many years.

### 4.3 Evolution of dietary preference and fang morphology

Burrowing asps and collared snakes have unique ecologies, particularly in terms of dietary preferences. *Atractaspis* in particular have very distinctive fangs (solenoglyphous fangs, similar to viperids) that have made their taxonomic history complicated (e.g., previously classified as viperids) [25, 31, 94]. The fangs of *Homoroselaps* resemble fangs of elapids more than vipers. In contrast, aparallactines tend to have rear fangs (Figs 3 and 6) [18, 25, 29–30]. Our ancestral-state reconstruction analysis of fang morphology suggested a rear fang ancestor for all collared snakes and burrowing asps (Aparallactinae and Atractaspidinae). Most lamprophiids are either rear fanged or lack fangs [25]. Our analyses also recovered dietary generalization as an ancestral-state for atractaspidines and aparallactines. Both of these conditions support the hypothesis proposed by Underwood and Kochva [18], which postulated that collared snakes and burrowing asps likely had a *Macrelaps*-like ancestor (large and rear fanged) that foraged above ground or in burrows of other organisms, and these taxa subsequently evolved into more specialized forms with specialized diets. Several aparallactines are dietary specialists [25, 31], that feed on the following: *Aparallactus* specialize on centipedes and possibly other invertebrates like earthworms; *Chilorhinophis* and *Amblyodipsas* consume snakes and other small, fossorial reptiles; *Polemon* are ophiophagous [25, 31, 95], but may occasionally consume other squamate prey items; *Macrelaps* consume reptiles, amphibians, and rarely mammals [25]; and *Xenocalamus* consume amphisbaenians [25, 31].

Unlike several aparallactines, *Atractaspis* are dietary generalists that consume a diverse variety of squamates, rodents (particularly nestling rodents), and occasionally amphibians [25, 31, 33, 52, 96–100]. The venom glands of *Atractaspis* are anatomically distinct from those of other front-fanged snakes such as viperids and elapids, because atractaspidines lack a distinct accessory gland and the presence of mucous-secreting cells at the end of each serous tubule [32, 101–103]. Similar to two other front-fanged snake groups (Elapidae and Viperidae), elongated venom glands have evolved within *Atractaspis* from western and northern African, and western Asia/Middle East species. These glands may be up to 12 cm long in *A*. *engaddensis* and 30 cm long in *A*. *microlepidota* [32]. Phylogenetically, *Atractaspis* is clearly partitioned according to venom gland length and geographic distribution (Figs 1 and 2). The purpose of these anatomical adaptations are unclear, although it is possible that they evolved to influence venom yield, as in *Calliophis bivirgatus* (Elapidae) [32]. The unique viper-like front fangs of *Atractaspis* may have evolved to facilitate the predation of rodent nestlings or squamates in tight burrows. Preying on animals in tight burrows limits mobility of the predator, because the body of the prey item can serve as a physical barrier, stopping the predator from further pursuit. Many lizards can detach their tails if a predator grabs the tails from behind. Shine et al. [31] postulated that it would be advantageous for a predator to push past the tail and envenomate or seize the prey by the body, a scenario ideal for *Atractaspis*. Deufel and Cundall [33] hypothesized that the evolution of the front fang in *Atractaspis* was likely the result of the following advantages: (1) greater envenomation efficiency resulting from the longer fangs; (2) closed mouth venom delivery system, allowing envenomation during head contact with any part of the prey; (3) capacity to quickly envenomate and release prey; and (4) potential for effective defense against adult rodents. Most prey consumed by *Atractaspis* (amphisbaenians, fossorial skinks, typhlopid snakes) [25] are also consumed by other atractaspidines and aparallactines, including *Amblyodipsas*, *Chilorhinophis*, *Homoroselaps*, *Macrelaps*, *Polemon*, and *Xenocalamus* [25, 31, 97]. These observations suggest that squamate prey are consumed across all atractaspidine and aparallactine genera, and therefore, they may not be the only selective force driving the evolution of the unique fang in *Atractaspis*. However, rodents and other mammals are not commonly preyed on by other burrowing asps and collared snakes [31, 104]. Deufel and Cundall [33] stated that it is unlikely that mammalian prey alone drove the evolution of a moveable front fang in *Atractaspis*, but the success and wide distribution of this genus may be partially attributed to mammalian prey. Unlike aparallactines, *Atractaspis* can quickly envenomate and dispatch all rodents in a nest [33]. A rear fang condition would require the snake to bite, hold and chew on every prey item, which is undoubtedly a more energetically costly form of envenomation compared with the predatory behavior of *Atractaspis*. Interestingly, in a feeding experiment, *Atractaspis* never attempted to ingest snake prey until the prey stopped reacting to fang pricks [33]. This observation suggests that *Atractaspis* will not risk injury until prey are completely immobilized. The unique fang and predatory behavior of *Atractaspis* has its functional trade-offs; *Atractaspis* lack large mandibular and maxillary teeth that allow snakes to quickly consume prey [33], and therefore, they take longer to ingest prey items. Because *Atractaspis* forage, kill, and consume prey in the soil and below the surface, there were likely no negative selective pressures acting against slow ingestion of prey. Because they are fossorial, *Atractaspis* may be relatively safe from predators while feeding, which is when non-fossorial snakes may be vulnerable to predation or attacks from other animals [25, 33].

Results from this study indicate that the rear-fang condition can cover a wide variety of dietary specializations. But this condition is not ubiquitous among aparallactines. *Aparallactus modestus* clearly lacks enlarged fangs (Figs 5 and 6), but previous studies have found venom glands in this taxon [105]. Additionally, the venom gland of *A*. *modestus* is reported to differ from the venom gland of *A*. *capensis*, but further details of the discrepancies were not discussed [32, 105, 106]. Interestingly, this species may prey on earthworms rather than centipedes (II pers. obs. [30]), explaining the loss of a rear-fang condition, which is present in all other *Aparallactus* species used for this study, including *A*. *niger*, the sister species to *A*. *modestus* (Figs 5 and 6).

*Polemon* fangs are not easily classified. The fangs of *Polemon* are located on the anterior half of the maxilla, rather than the more typical posterior end (Figs 5 and 6). These fangs are large and deeply grooved, and resemble a fixed front-fang condition, but yet they are positioned behind one or two smaller maxillary teeth. The ophiophagous diet of *Polemon* likely influenced the evolution of a front-fang condition in this genus. *Polemon* are known to prey on large and formidable snake prey, which can rival the predator in size [35, 48, 95, 107]. With large, deeply grooved fangs positioned on the anterior side of the maxilla, *Polemon* can quickly envenomate and kill relatively large and powerful prey (snakes) more effectively than they would with a rear-fang condition like *Aparallactus*. Snakes with rear fangs must typically chew in a forward orientation until the rear fang can penetrate the flesh of the prey item [25]. Several front-fanged, elapid genera prey heavily on snakes (e.g., *Micrurus* and *Ophiophagus*). The front-fang condition may be a favorable trait to feed on snakes, in order to immobilize and kill more quickly.

In *Xenocalamus*, similar selective pressures (e.g., tight burrow foraging) that led to the evolution of fang and predatory behaviors in *Atractaspis*, may have led to the evolution of its unique quill-shaped snout [31]. Unlike *Amblyodipsas polylepis*, *Xenocalamus* possess relatively large maxillary teeth that gradually increase in size from the anterior to posterior side of the maxilla (Figs 3 and 5). This trait seems advantageous to improve their grasp of amphisbaenian prey.

It is not surprising that the rear fang and dietary generalist conditions were recovered as the ancestral-state condition for both atractaspidines and aparallactines, considering many lamprophiids are dietary generalists [25, 30]. Collared snakes and burrowing asps seem to have experienced the opposite of niche conservatism as results herein indicated that foraging behaviors and diet have heavily and rapidly influenced the evolution of fang morphology, dietary specializations, and snout shape. In collared snakes (aparallactines), dietary specializations seem to have shaped variation (and loss) of fangs and snout shape, particularly for *Aparallactus*, *Polemon*, and *Xenocalamus*. These genera tend to have more specialized diets than *Macrelaps*, *Chilorhinophis* and *Amblyodipsas*, all of which possess more typical rear fangs (Figs 3 and 5) [25, 30–31]. A fundamental controversy in snake evolution is whether front and rear fangs share the same evolutionary and developmental origin. Burrowing asps and collared snakes possess all known types of snake dentition (no fang, rear fang, fixed front fang, and moveable front fang). Our results lend credence to the hypothesis that rear fangs and front fangs share a common origin [94]. Our results also indicated that snake dentition, specifically alethinophidian groups such as atractaspidines and aparallactines, may be highly plastic within relatively short periods of time to facilitate foraging and life history strategies.

## Supporting information

S1 File Settings for high-resolution CT scans and DOI numbers for supporting files on the Morphosource website, in Microsoft Excel format. (XLSX)
