# Correction: Evolutionary history of burrowing asps (Lamprophiidae: Atractaspidinae) with emphasis on fang evolution and prey selection

**DOI:** 10.1371/journal.pone.0217035

**Published:** 2019-05-13

**Authors:** The PLOS ONE Staff

In the Funding statement, the author initials associated with Flora Fauna & Man, Ecological Services Ltd. (FFMES) are incorrect. The correct Funding statement is: This work was supported by the Percy Sladen Memorial Fund, an IUCN/SSC Amphibian Specialist Group Seed Grant, K. Reed, M.D., research funds from the Department of Biology at Villanova University, and UTEP (all to EG), National Science Foundation grant DEB-1145459 (to EG and KJ), National Geographic Research and Exploration Grant no. 8556–08 (to EG), National Geographic Okavango Wilderness Project no. EC0715-15 (to WC), Belgian National Focal Point to the Global Taxonomy Initiative (to ZTN). MOR is supported by the Gorongosa Restoration Project and the Mozambican Departamento dos Serviços Cientificos (PNG/DSCi/C12/2013; PNG/DSCi/C12/2014; PNG/DSCi/C28/2015). The University of Texas at El Paso (UTEP) Border Biomedical Research Center (BBRC) Genomic Analysis Core Facility is acknowledged for services and facilities provided. This core facility is supported by grant 5G12MD007592 to the (BBRC) from the National Institutes on Minority Health and Health Disparities (NIMHD), a component of the National Institutes of Health (NIH). Marius Burger received payment as a herpetologist consultant with Flora Fauna & Man, Ecological Services Ltd. (FFMES). The funder provided support in the form of salaries for one author [Marius Burger], but did not have any additional role in the study design, data collection and analysis, decision to publish, or preparation of the manuscript. The specific roles of these authors are articulated in the ‘author contributions’ section. The funders had no role in study design, data collection and analysis, decision to publish, or preparation of the manuscript.

In the Competing Interests statement, the author initials are incorrect. The correct Competing Interests statement is: Marius Burger is affiliated with Flora Fauna & Man, Ecological Services Ltd. This does not alter our adherence to PLOS ONE policies on sharing data and materials.

There is an error in the genus name in reference 35. The correct reference is: Portillo F, Branch WR, Tilbury CR, Nagy ZT, Hughes DF, Kusamba C, et al. A cryptic new species of *Polemon* (Squamata: Lamprophiidae, Aparallactinae) from the miombo woodlands of Central and East Africa. Copeia, 2019; 107: 22–35. DOI: 10.1643/CH-18-098.

There is an error in the genus name in reference 74. The correct reference is: Portillo, F, Greenbaum E, Menegon M, Kusamba C, Dehling JM. Phylogeography and species boundaries of *Leptopelis* (Anura: Arthroleptidae) from the Albertine Rift. Mol. Phylogenet. Evol., 2015; 82: 75–86. DOI: 10.1016/j.ympev.2014.09.024.

There are errors in the genus names in reference 76. The correct reference is: Medina, MF, Bauer AM, Branch WR, Schmitz A, Conradie W, Nagy ZT, et al. Molecular phylogeny of *Panaspis* and *Afroablepharus* skinks (Squamata: Scincidae) in the savannas of sub-Saharan Africa. Mol. Phylogenet. Evol., 2016; 100: 409–423. DOI: 10.1016/j.ympev.2016.04.026.

There is an error in the genus name in reference 78. The correct reference is: Greenbaum, E, Dowell Beer SA, Wagner P, Anderson CG, Villanueva CO, Malonza P, et al. Phylogeography of Jackson’s Forest Lizard *Adolfus jacksoni* (Sauria: Lacertidae) reveals cryptic diversity in the highlands of East Africa. Herpetol. Monogr., 2018; 32: 51–68. DOI: 10.1655/HERPMONOGRAPHS-D-18-00005.1.

The publisher apologizes for the errors.
